# Cell death in glioblastoma and the central nervous system

**DOI:** 10.1007/s13402-024-01007-8

**Published:** 2024-11-06

**Authors:** Kyle Malone, Eric LaCasse, Shawn T. Beug

**Affiliations:** 1 Apoptosis Research Centre, Children’s Hospital of Eastern Ontario Research Institute, 401 Smyth Road, Ottawa, ON K1H 8L1 Canada; 2 Department of Biochemistry, Microbiology and Immunology, University of Ottawa, 451 Smyth Road, Ottawa, ON K1H 8M5 Canada; 3 Centre for Infection, Immunity and Inflammation, University of Ottawa, 451 Smyth Road, Ottawa, ON K1H 8M5 Canada; 4 Ottawa Institute of Systems Biology, University of Ottawa, 451 Smyth Road, Ottawa, ON K1H 8M5 Canada

**Keywords:** Apoptosis, Necroptosis, Glioblastoma, Inhibitor of Apoptosis, Astrocytes, Oligodendrocytes

## Abstract

Glioblastoma is the commonest and deadliest primary brain tumor. Glioblastoma is characterized by significant intra- and inter-tumoral heterogeneity, resistance to treatment and dismal prognoses despite decades of research in understanding its biological underpinnings. Encompassed within this heterogeneity and therapy resistance are severely dysregulated programmed cell death pathways. Glioblastomas recapitulate many neurodevelopmental and neural injury responses; in addition, glioblastoma cells are composed of multiple different transformed versions of CNS cell types. To obtain a greater understanding of the features underlying cell death regulation in glioblastoma, it is important to understand the control of cell death within the healthy CNS during homeostatic and neurodegenerative conditions. Herein, we review apoptotic control within neural stem cells, astrocytes, oligodendrocytes and neurons and compare them to glioblastoma apoptotic control. Specific focus is paid to the Inhibitor of Apoptosis proteins, which play key roles in neuroinflammation, CNS cell survival and gliomagenesis. This review will help in understanding glioblastoma as a transformed version of a heterogeneous organ composed of multiple varied cell types performing different functions and possessing different means of apoptotic control. Further, this review will help in developing more glioblastoma-specific treatment approaches and will better inform treatments looking at more direct brain delivery of therapeutic agents.

## Introduction

Programmed cell death (PCD) refers to a multitude of processes through which cells are cleared during the lifespan of an organism. PCD proceeds through defined biochemical and identifiable genetic pathways that are distinguishable from accidental forms of necrotic cell death [1]. As of 2018, eleven forms of PCD have been described in response to a variety of stimuli and conditions [2, 3], with apoptosis the first identified and most extensively studied [4]. Apoptotic cell death can be triggered by cell stress signals, such as growth factor withdrawal, radiation, excessive reactive oxygen species (ROS), and death ligand signaling through death receptors. These trigger the intrinsic (mitochondrial) and extrinsic (death receptor) apoptotic pathways, which converge on effector proteases of the caspase family. Activation of effector caspases result in blebbing of the cellular membrane, cell shrinkage, nuclear fragmentation and chromatin condensation. The resultant clearance of apoptotic bodies is mediated through phagocytosis by phagocytes, limiting inflammation [5]. Apoptosis is regulated through differential expression of pro- and anti-apoptotic proteins, necessitating a strong enough trigger to shift the balance and effect cell death. Regulation of cell death is significantly perturbed in cancer, leading to resistance of cancer cell death to apoptotic stimuli.

Glioblastoma (GBM) is the most common and lethal primary brain tumor, presenting an overall median survival of under 15 months with current treatments (surgical resection, radiotherapy (RT) and temozolomide (TMZ) chemotherapy) [6]. While the incidence of central nervous system (CNS) tumors is low, as a whole these tumors have the third highest mortality to incidence ratio of any human cancer and the highest average treatment costs [7, 8]. Advanced age is the only known risk factor for primary GBM, with the greatest incidence and mortality rate in patients over 60 for whom it is near uniformly fatal [9, 10]. Pediatric GBM is rare and given the lack of clinically effective therapies, these patients are treated identically to adult cases with only slightly better outcomes [11–13]. GBMs are thought to originate from either neural stem cells (NSCs) in the subventricular zone (SVZ), which acquire oncogenic driver mutations and migrate away from the SVZ and develop into tumor [14], or from highly proliferative oligodendrocyte progenitor cells (OPCs) [15]. Deregulation of receptor tyrosine kinases (RTKs), as well as dysregulated RAS-MEK-ERK, PI3K-AKT, p53, and Rb pathways are all seen in a majority of GBM patients, and have been proposed as a necessity for gliomagenesis [16]. Within a tumor, glioma stem cells (GSCs), differentiated OPCs and astrocyte-like cells co-exist, providing each other with supportive cues [17] and joining into neuronal networks [18]. GSCs have also been shown to generate GBM-specific pericytes to control vasculature function [19]. Significant infiltration into the surrounding brain via vasculature or along white matter fibres [20, 21], intra- and intertumoral heterogeneity, necrosis, substantial angiogenesis, and resistance to apoptosis are all hallmarks of GBM [22].

GBMs recapitulate many features of CNS development and injury responses [23]. It is thus important to examine cell death control mechanisms within the CNS, how each cell type uniquely regulates PCD, how differences of PCD in CNS cells contribute to a baseline heterogeneity of GBM apoptotic resistance, and of PCD pathway alterations associated with GBM oncogenic transformation. In the scope of this review, we refer GBM to be tumor cells that are distinct from other cell types recruited to support the GBM microenvironment. While the gold standard for oncology research is analysis of primary tumor tissue, much of the presented information derives from publications of extensive in vitro analysis using immortalized GBM cell lines. Of the cells in the CNS, comparisons to GBM will be made to NSCs/NPCs, astrocytes, neurons, oligodendrocytes and OPCs. These cell types encompass the likely origin of GBM cells and their major differentiated progeny [24]. While infiltrating immune cells and endothelial cells are crucial for GBM development and biology, their discussion is outside the scope of this review. Similarly, microglia and macrophages are central to all aspects of GBM biology [25, 26] and we have examined their apoptotic sensitivity in a GBM context elsewhere [27]. Given the increasing attention paid to the extrinsic pathway in the context of inflammation and immunotherapy, regulation of the death receptor apoptotic pathway, particularly by the Inhibitor of Apoptosis (IAP) proteins will be of particular focus, and potential unique vulnerabilities will be highlighted.

## Cell death pathways

### Intrinsic apoptotic pathway

**Fig. 1:**
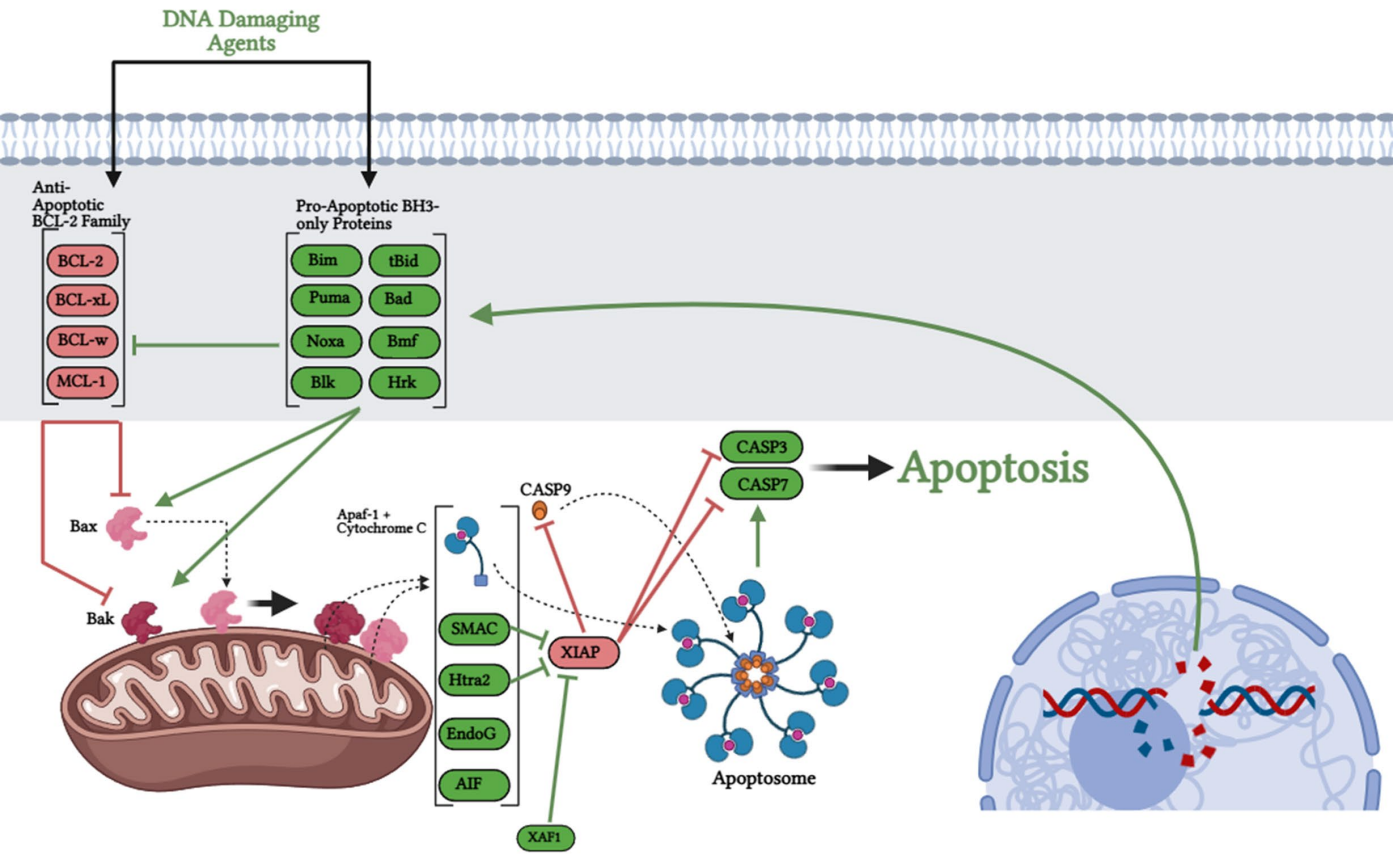
Intrinsic apoptotic pathway. DNA damaging agents including chemotherapy and radiation induce expression of pro-apoptotic BH3-only proteins, exceeding the balance with anti-apoptotic BCL-2 family proteins and permitting BAX and BAK oligomerization in the mitochondrial outer membrane. This outer membrane is consequently permeabilized, allowing release of pro-apoptotic factors including Apaf-1 and Cytochrome C, which form the apoptosome with CASP9 and cleave downstream effector CASP3 and CASP7. SMAC release from the mitochondria inhibits XIAP, a crucial step in permitting apoptosis

The intrinsic apoptotic pathway is initiated by intracellular cytotoxic stimuli and DNA damage, resulting in mitochondrial outer membrane permeabilization (MOMP; Fig. 1). This permeabilization is effected by the monomeric BCL-2 family proteins BAX and BAK. Upon exposure to an apoptotic signal, BAX translocates to the mitochondria; BAK is constitutively inserted in the mitochondrial outer membrane. Oligomerization results in the formation of a pore in the outer mitochondrial membrane [28–30], allowing for release of various apoptogenic proteins depending on the degree of permeabilization into the cytosol, such as cytochrome C (CYCS), second mitochondrial activator of caspases (SMAC), Htra serine peptidase 2 (HTRA2), endonuclease G, and apoptosis-inducing factor (AIF) [31, 32], representing for most cells the point of no return in the apoptotic cascade [2, 33]. CYCS-Apaf-1 oligomerizations forms the apoptosome [34–37], a CASP9 holoenzyme complex, which oligomerizes and activates procaspase 9 (pro-CASP9) monomers [36]. The resulting complex cleaves and activates the downstream amplifying effector caspases CASP3 and CASP7 [38, 39], in turn effecting apoptosis through cleavage of enzymes critical in DNA repair, such as poly-ADP ribose polymerase (PARP) [40, 41]. The regulation of MOMP is achieved through expression of pro- and anti-apoptotic BCL-2 family members. Anti-apoptotic members, which include BCL-2, BCL-xL, BCL-w, and MCL-1, can bind and sequester BAX and BAK [42, 43], preventing MOMP. Pro-apoptotic BH3-only proteins (BIM, BID, PUMA, BAD, NOXA, BMF, BLK, HRK) can bind and inactivate these anti-apoptotic proteins and, in the case of BID, BIM and PUMA, directly bind and activate BAX and BAK [44–47].

### Extrinsic apoptotic pathway

**Fig. 2:**
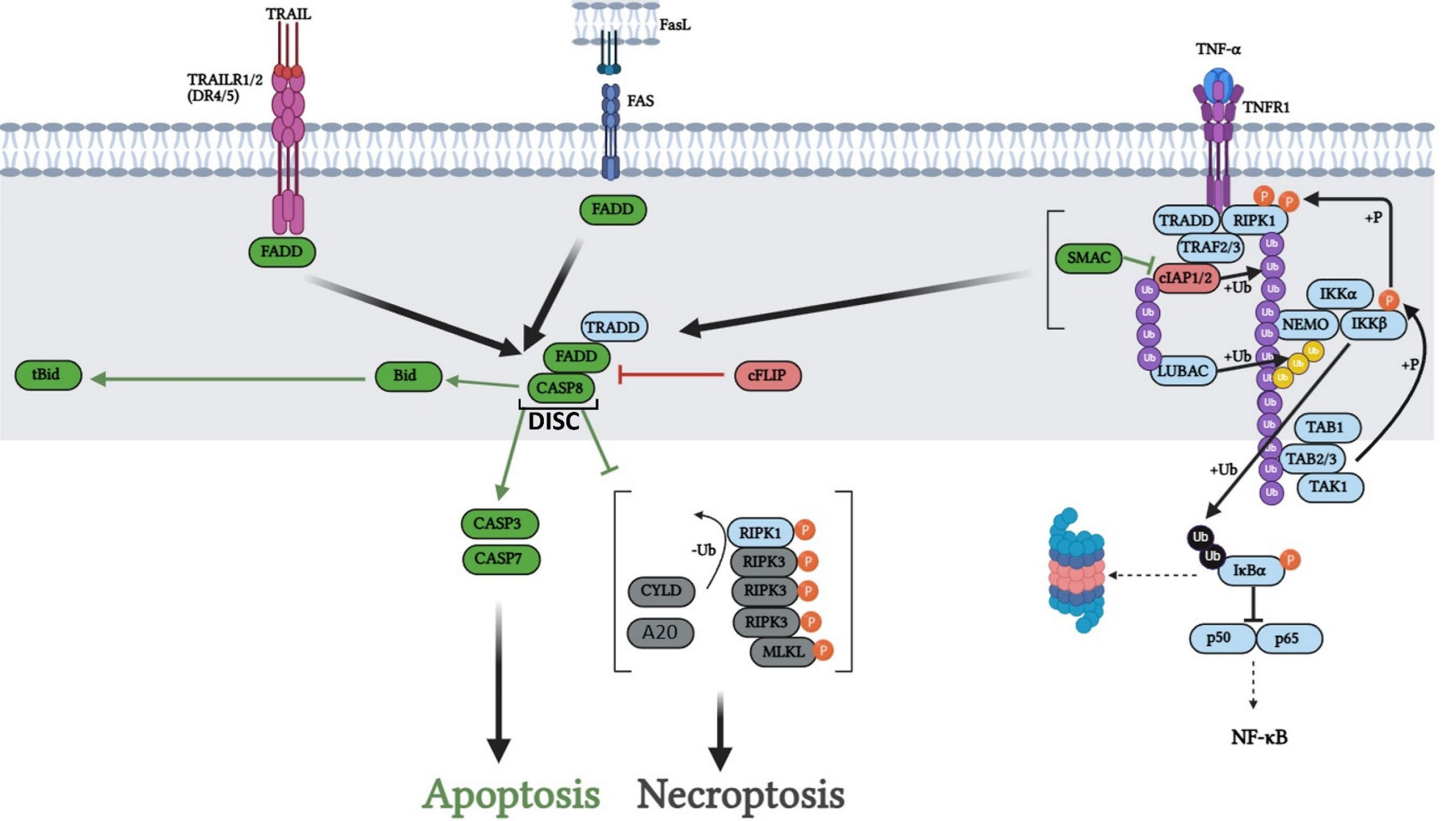
Death receptor apoptotic pathway. Engagement of TNFR superfamily death receptors (TRAILR1/2 (DR4/5), FAS, TNFR1) by their ligands (TRAIL, FasL, TNF-α) leads to FADD recruitment. FADD in turn recruits multiple pro-CASP8 monomers, forming the DISC/Faddosome/Ripoptosome. Pro-CASP8 cleavage to activated CASP8 leads to cleavage and activation of CASP3 and CASP7, effecting apoptosis and cleavage of Bid to tBiD, engaging the intrinsic apoptotic pathway. cFLIP binding FADD inhibits subsequent pro-CASP8 binding and DISC or DIC2 formation. In TNFR1 signaling, TRADD and RIPK1 are initially recruited following TNF-α binding. TRAF2/3 is subsequently recruited, and in turn recruits cIAP1/2 which ubiquitinate RIPK1. This ubiquitinated RIPK1 represents a scaffold for downstream signaling to activate IKK to degrade IκBα, permitting NF-κB activation. In the absence of cIAP1/2, lack of RIPK1 ubiquitination results in recruitment of FADD by TRADD and formation of the DIC2. Engagement of death receptors in the absence of CASP8 results in CYLD or A20 deubiquitination of RIPK1 and RIPK3 and consequent RIPK1-RIPK3-MLKL dependent necroptosis

The extrinsic apoptotic pathway is initiated by engagement of certain members of the tumor necrosis factor receptor (TNFR) superfamily (Fig. 2). These receptors are characterized by the presence of cytoplasmic death domains (DD), including Fas cell surface death receptor (FAS), TNF-related apoptosis inducing ligand receptor 1/2 (TRAILR1/2, also referred to as Death Receptor 4/5 (DR4/5), respectively), and TNFR1. In the case of FAS- and TRAILR1/2-induced death, binding of its ligand FASL or TRAIL to the receptor results in the recruitment of the adaptor protein FAS-associated protein with death domain (FADD). FADD recruits pro-CASP8, forming the death inducing signaling complex (DISC), also referred to as the Faddosome. Autocatalytic cleavages activate CASP8, resulting in cleavage of effector caspases and thereby apoptosis [48–53]. In some cases, CASP8 further cleaves BID to truncated BID (tBID), allowing for initiation of the mitochondrial pathway [54].

The binding of TNF-α to TNFR1 normally leads to engagement of the classical Nuclear Factor kappa B (NF-κB) pathway, which leads to expression of survival and inflammatory genes [55, 56]. However, in the absence of cellular IAP 1 and 2 (cIAP1 and cIAP2), the TNF-α signal switches from a pro-survival to a pro-death response. Mechanistically, the loss of cIAP1/2 leads to lack of receptor interacting protein kinase 1 (RIPK1) ubiquitination, which leads to FADD associating with the DD of TNFR1. This then recruits and activates proCASP8, forming the death inducing complex 2 (DIC2) (also referred to as the ripoptosome) and effecting downstream apoptosis [57]. The presence/absence or inhibitory status of CASP8 determines the mode of cell death, as CASP8 protease activity can cleave RIPK1, RIPK3, and cylindromatosis (CYLD), preventing the formation of the necroptosome [58–60]. The deubiquitinating enzyme A20 plays redundant roles to CYLD in deubiquitinating RIPK1 and RIPK3 in NF-κB and necroptotic signaling, with cell type-specific functions [61]. Without CASP8, CYLD or A20 deubiquitinates RIPK1, which then recruits and interacts with RIPK3. Activated RIPK3 phosphorylates mixed lineage kinase domain-like pseudokinase (MLKL); this RIPK1-RIPK3-MLKL complex is the necroptosome. Activated MLKL oligomerizes and creates membrane pores leading to substantial ion flux, release of inflammatory damage associated molecular patterns, and necroptotic cell death [62–65].

Aside from cIAP1/2 action as a switch between cell survival and cell death in TNF-α signaling, the regulation of the extrinsic apoptotic pathway is primarily achieved through the actions of CASP8 (or similar CASP10), FADD-like apoptosis regulator (cFLIP), and X-linked IAP (XIAP). cFLIP structurally resembles CASP8, binding FADD and preventing DISC or DIC2 assembly. Further, cleavage products of cFLIP activate cell survival pathways (AKT, JNK, WNT, NF-κB) via interactions with TRAF2 and RIPK1 [66–68]. The direct caspase-inhibitory effects of XIAP also determine whether extrinsic apoptosis is type 1 or type 2. Type 1 cells die through the caspase cascade initiated by pro-CASP8 processing and activation, while type 2 cells require the further cleavage of BID to tBID by CASP8 and engagement of the mitochondrial CASP9-dependent apoptotic pathway. This is due to altered ratios between effector caspases and XIAP, with cells possessing high XIAP showing compensatory increases upon exposure to death ligands, requiring SMAC release and IAP inhibition to effect apoptosis [69, 70].

Within the CNS, resistance to death ligands is also maintained by the protein anti-apoptotic phosphoprotein enriched in astrocytes 15 kDa (PEA-15). PEA-15 contains an N-terminal death effector domain that inhibits DISC formation through FADD and proCASP8 binding, blocking death receptor-induced apoptosis [71, 72]. PEA-15 is primarily expressed in the brain (neurons, astrocytes, and NSCs/NPCs) [73]. Major posttranslational control is achieved through differential phosphorylation at the Ser104 and Ser116 residues in the C-terminus through the actions of PKC (Ser104), CAMKII, and AKT (Ser116) [74–76]. These phosphorylations result in allosteric modifications favouring distinct binding partners [77]. CAMKII is highly expressed in the CNS [78]. Dephosphorylation at the Ser104 site is effected by protein phosphatase 2 A (PP2A) [71, 79], while phosphatase and tensin homolog on chromosome 10 (PTEN) modulates Ser116 phosphorylation via its regulation of Akt activation [80]. PP4 also acts to directly dephosphorylate Ser116 of PEA-15 [81]. Unphosphorylated PEA-15 can bind ERK, anchoring it in the cytoplasm, thereby inhibiting its nuclear translocation and downstream transcription of target genes. Phosphorylation at Ser104 blocks the ability of PEA-15 to sequester ERK. Phosphorylation at Ser116 allows for PEA-15 to inhibit DISC formation and prevent apoptosis [82–84]. As a result, PEA-15 is able to control proliferation, migration and apoptosis depending on phosphorylation status [85], with phosphorylation also stabilizing the protein [75]. PEA-15 is decreased in multiple neurodegenerative conditions, setting the stage for progressive cell death [79, 86, 87].

## Regulation of GBM cell death through the intrinsic apoptotic pathway

### Intrinsic apoptotic regulation in GBM

Neurons can form synapses with GBM cells. Neuroligin-3 (NLG3), a neuroligin responsible for synapse construction and maintenance, plays a key role in neuron to GBM communication, GBM progression, and stimulation of oncogenic signaling pathways. Among these, exposure of GBM cells to NLG3 significantly increases phosphorylated AKT levels [18]. Relatedly, the majority of GBMs display inactivation of PTEN (an inhibitor of PI3K-AKT signaling), either through deletion, inactivating mutation, or methylation [88, 89]. High and unregulated PI3K-AKT signaling results in significant alterations to mitochondrial apoptotic cascade members; in many cancers, overactivation of AKT results in resistance to numerous apoptotic stimuli [90]. Reintroduction of PTEN results in reduced viability and proliferation in several human GBM lines [91]. The pro-apoptotic BH3-only protein BAD is phosphorylated and inactivated by AKT [92], freeing BCL-2 and BCL-xL to inhibit further apoptotic signaling. Pro-CASP9 is also phosphorylated and inhibited by AKT [90]. Astrocyte-GBM interactions via gap junctions or secreted factors increase NF-κB, JAK/STAT and MAPK/JNK signaling, resulting in increased BCL-2 and IAP expression and reduced BAX and consequent resistance to radiation and chemotherapy [93–99]. Preclinical investigations looking at inhibiting overexpressed BCL-2 family members in GBM are summarized in Table 1 and clinical trials looking at these BH3-mimetic compounds for recurrent GBM are summarized in Table 2.

The pro-survival pathway mediated by the transcription factor NF-κB is constitutively activated at a high level in GBM, which is linked to AKT activity. NF-κB activation via AKT phosphorylation of IKKα results in transcription of multiple anti-apoptotic genes, including BCL-2 family members, cIAP1/2, XIAP and Survivin, of which cIAP2 is the strongest target. Inhibition of AKT via induction of PTEN expression or chemical inhibition of PI3K results in significant decreases in NF-κB activity [90, 100–102]. Overactivated Notch signaling is a common feature of GBMs, and crosstalk with the AKT pathway further promotes apoptosis resistance [103, 104]. AKT (among other cell survival pathways) is also activated by RTKs, which are often significantly overactivated in GBM through a combination of either gene amplification, ligand and receptor overexpression, or, mutations conferring constitutive activation such as EGFRvIII [22]. EGFRvIII overexpression correlates with increased expression of anti-apoptotic BCL-2 family members [22]. Numerous clinical trials targeting individual RTKs (EGFR, PDGFR, IGFR, FGFR, VEGFR) have been completed or are ongoing; however little to no benefit to progression free survival has been noted [105], suggesting a need to target multiple RTKs simultaneously.

Dysregulated AKT and NF-κB signaling heavily skew intrinsic pathway regulators toward an anti-apoptotic phenotype. APAF-1 and pro-CASP9 are expressed at significantly lower levels in GBM relative to HeLa cells. While expression across tumors was variable, high pro-CASP3 levels were associated with longer progression-free survival times; APAF-1, SMAC or XIAP showed no such correlation [106]. TMZ-sensitive GBM cell lines show significantly higher expression of proCASP3 compared to resistant GBM cell lines [106]. BCL-2 family members BCL-2, BCL-xL and MCL-1 are heterogeneously expressed, but generally overexpressed in GBMs relative to normal brain [107], and especially high in GSCs [108]. Conversely, BAX levels are significantly reduced [107]. High STAT3 signaling in GSCs increases expression of BCL-2, BCL-xL, MCL-1 [109, 110] and Survivin [111]. High expression of multiple BH3-only proteins correlates with better overall survival, however no individual protein correlated with improved prognosis. High phosphorylation of BAD and BIM were associated with low overall survival [112], a poor prognostic indicator given that AKT can effect this phosphorylation and AKT is highly overactivated in GBM.

**Table 1:** Direct apoptotic pathway targeting agents in preclinical GBM

Cell line/model	Agent	Target	Combinatorial agents	Outcome	Ref.
SNB19 (H, Imm) SNB75 (H, Imm) SF268 (H, Imm) SF295 (H, Imm) SF539 (H, Imm) U251 (H, Imm)	**BH3 mimetics**: S63845	**BCL-2 targets**: MCL-1	JQ1 (BET family bromodomain protein inhibitor) TMZ Erastin (system xCT inhibitor)	• Single agent apoptosis induction - Synergy of combinations of BH3 mimetics and TMZ • BAX and BAK depletion inhibit killing efficacy of BH3 mimetics but not TMZ & JQ1 • A1331852 penetrates brain and brain tumor • BH3 mimetics aid in ferroptosis induction	[113]
	A1331852	BCL-xL			
	Venetoclax/ABT-199	BCL-2			
Patient GSCs (H, P) Fresh, resected GBM tissue (H, P) U87MG (H, Imm)	**BH3 mimetics**: A1331852	**BCL-2 targets**: BCL-xL	RT TMZ	• GSCs and stem cell enriched U87 cells sensitive to MCL-1 and BCL-xL inhibition, minimally BCL-2 inhibition • No synergism with RT and TMZ • MCL-1 required for tumor growth • MCL-1 and BCL-xL dual inhibition induces substantial GSC apoptosis • Dual MCL-1 and BCL-xL targeting causes apoptosis of primary human GBM ex vivo • BIM upregulation following BCL-xL inhibition underlies sensitivity to subsequent MCL-1 targeting	[114]
	S63845	MCL-1			
	Venetoclax/ABT-199	BCL-2			
	Navitoclax/ABT-737	BCL-2, BCL-xL & BCL-w			
A172 (H, Imm) YKG1 (H, Imm) LN18 (H, Imm) U87MG (H, Imm)	**BH3 mimetic**: BAU-243	**BCL-2 targets**: BCL-2		• ABT-199 and BAU-243 induce apoptosis, inhibit sphere forming capacity dependent on high BCL-2 expression • BAU-243 induces autophagic cell death regardless of BCL-2 expression • BAU-243 increases survival, reduces tumor growth in mice bearing U87MG	[115]
	Venetoclax/ABT-199	BCL-2			
U87 (H, Imm) LN229 (H, Imm) T98G (H, Imm) GBM6 (H, P) GBM14 (H, P) GBM39 (H, P) GSCs (NCH644, NCH421k; H, Imm)	**BH3 Mimetics**: ABT-263	**BCL-2 targets**: BCL-xL, BCL-2 & BCL-w (not MCL-1)	JQ1 & OTX015 (BET inhibitors)	• Synergistic apoptosis induction following targeting BCL-2 alone and in combination with cMyc inhibition regardless of immortalized, primary or GSC cell type • OTX015 and ABT-263 reduce LN229 size in vivo • MCL-1 is resistance factor for ABT-263– JQ1 increases Noxa, inhibiting MCL-1 and enhancing efficacy of ABT-263	[116]
	Obatoclax/GX15-070	Pan BCL-2			
U87MG (H, Imm) U251MG (H, Imm) NCH89 (H, P) NCH156 (H, P)	**BH3 Mimetics**: ABT-737	**BCL-2 targets**: BCL-2, BCL-xL	Vincristine Etoposide TRAIL	• MCL-1 is resistance factor for ABT-737. Knockdown increases sensitization and apoptosis induction in immortalized, primary and stem cell enriched GBMs • ABT-737 increases survival of U251MG-bearinig mice • ABT-737 enhances efficacy of vincristine, etoposide and TRAIL	[117]
U87MG (H, Imm)	**BH3 Mimetics**: MIM1	**BCL-2 targets**: MCL-1 specific	TMZ	• MIM1 causes significant U87MG apoptosis, synergizes with TMZ	[118]
U251MG (H, Imm) U373 (H, Imm) U343 (H, Imm) U87 (H, Imm)	**BH3 Mimetic**: Gossypol/AT-101	**BCL-2 targets**: Pan BCL-2	YM-1 (HSP70/BAG3 inhibitor)	• Autophagy inhibition by BAG3 blockade enhances AT-101 and ABT-737 apoptosis induction	[119]
	ABT-737	BCL-2, BCL-xL			
T98G (H, Imm)	**BH3 Mimetic**: ABT-737	**BCL-2 targets**: BCL-2, BCL-xL		• Astrocytes and GBM cells are sensitive to ABT-737 and MIM1	[120]
	MIM1	MCL-1			
T98G (H, Imm) U87MG (H, Imm) DBTRG-05MG (H, Imm) NMC-G1 (astrocytoma, H, Imm)	**BH3 Mimetic**: Gossypol/AT-101	**BCL-2 targets**: Pan BCL-2		• GBM cells undergo G2/M-phase cell cycle arrest following gossypol treatment. Both autophagic and apoptotic cell death observed	[121]
LN229 (H, Imm) A172 (H, Imm) U87MG (H, Imm)	**BH3 Mimetic**: Venetoclax	**BCL-2 target**: BCL-2	TMZ BV6 (SMC)	• BCL-2 and cIAP2 increased following TMZ treatment • BV6 and Venetoclax cotreatment act to increase TMZ-induced apoptosis and as senolytics depending on treatment time point	[122]
GBM6 (H, P) GBM10 (H, P, recurrent GBM) GBM12 (H, P) GBM39 (H, P) GBM76 (H, P, recurrent GBM) GBM123 (H, P, recurrent GBM) GBM164 (H, P) GBM196 (H, P)	**BH3 Mimetis**: Navitoclax	**BCL-2 targets**: BCL-2, BCL-xL, BCL-w	TMZ RT	• RT induces senescence in patient-derived GBM, susceptible to senolytics • Navtioclax, A1331852 and A1155463 kill senescent GBM cells post-RT. No effect of venetoclax • BCL-xL inhibition significantly increased apoptosis of radiated and/or TMZ-treated GBM cells, dependent on time of treatment	[123]
	A1331852	BCL-xL			
	A1155463	BCL-xL			
	Venetoclax	BCL-2			
U87MG (H, Imm)	**BH3 Mimetics**: Hypericin	**BCL-2 targets**: Pan BCL-2		• Hypericin > Gossypol > ABT-263 for BCL-2 inhibition • Gossypol > Hypericin > ABT-263 for MCL-1 inhibition • Hypericin did not affect viability of U87MG cells, similar to ABT-263. Gossypol substantially reduced viability in dose-dependent manner. • Upregulated MCL-1 post-treatment conferred resistance to Hypericin but not Gossypol, consistent with each agents’ MCL-1 inhibition capacity	[124]
	Gossypol	Pan BCL-2			
	ABT-263	BCL-2, BCL-xL			
GSC12 (H, P)	**BH3 Mimetic**: Navitoclax	**BCL-2 targets**: BCL-2, BCL-xL & BCL-w	Vorinostat (HDAC inhibitor) Trametinib (MEK inhibitor)	• MCL-1 is most highly expressed BCL-2 family protein • Combination Vorinostat + Navitoclax or S63845 significantly increased CC3 levels in GSCs. No effect of Navitoclax or S63845 alone	[125]
	S63845	MCL-1			
LN229 (H, Imm) U87 (H, Imm) U373 (H, Imm) NCH421K (GSC, H, Imm) NCH644 (GSC, H, Imm) NCH690 (GSC, H, Imm) GS9-6 (GSC, H, P)	**BH3 Mimetic**: ABT-263	**BCL-2 targets**: BCL-2, BCL-xL	GDC-0941 (PI3K inhibitor)	• GBM cells express high MCL-1, phosphorylated/inactivated BAD • ABT-263 reduced LN229 viability in dose-dependent fashion, synergizes with GDC-0941 • No effect of ABT-263 on U373 cells alone. Significant reductions in combination with GDC-0941 • ABT-263 or ABT-199 + GDC-0941 reduces GBM/GSC sphere growth and viability • Part of mechanism of action is GDC-0941 reduction in MCL-1 levels and reduced BAD phosphorylation • siRNA knockdown of MCL-1 sensitizes GBM to ABT-263 and ABT-199	[126]
	Venetoclax/ABT-199	BCL-2			
GSCs, labelled MGG6, MGG8, MGG23, 157, 83 and BT07 (H, P)	**Smac Mimetic**: Birinapant (D)	**Targets**: cIAP1, cIAP2, XIAP	UNC1999 (EZH2 inhibitor) GSK343 (EZH2 inhibitor) GSK126 (EZH2 inhibitor)	• GSCs resistant to birinapant in vitro and in vivo, even when combined with RT • Birinapant increased cancer stem cell content of GSCs via sustained NF-κB and STAT3 activity • cIAP2 increased by addition of TNFα • SMC combined with EZH2 inhibition significantly reduces GSC viability	[127]
	LCL161 (M)	cIAP1, cIAP2, XIAP			
U251 (H, Imm) U87 (H, Imm)	**Smac Mimetic**: Birinapant (D)	**Targets**: cIAP1, cIAP2, XIAP	RT	• Birinapant enhances radiosensitivity of GBM cells in vitro and in vivo, significantly extending mouse survival in U251-bearing animals	[128]
A172 (H, Imm) T98G (H, Imm) U87MG (H, Imm)	**Smac Mimetic**: BV6 (D)	**Targets**: cIAP1, cIAP2, XIAP		• BV6 had little effect on GBM or astrocyte viability, instead increasing CCL2 expression and migratory behaviours	[129]
A172 (H, Imm) T98G (H, Imm) U87MG (H, Imm) GBSC1, GBSC2, GBSC3 (GSCs, H, P) Primary patient GBM cells, unlabelled	**Smac Mimetic**: BV6 (D)	**Targets**: cIAP1, cIAP2, XIAP	RT	• BV6 significantly increased GBM cell death in combination with irradiation. Finding consistent between immortalized, primary and GSC-enriched cultures	[130]
U87MG (H, Imm) A172 (H, Imm) U118 (H, Imm) D54 (H, Imm– Now considered A172-derived)	**Smac Mimetic**: BV6 (D)	**Targets**: cIAP1, cIAP2, XIAP	TMZ Carmustine/BCNU TRAIL	• BV6 significantly reduces GBM cell viability in combination with TMZ or Carmustine • BV6-TMZ cotreatment induces DISC formation. • Effect not due to autocrine or paracrine TNFα signalling • TRAIL induced significant apoptosis in A172 cells as single agent	[131]
A172 (H, Imm) T98G (H, Imm)	**Smac Mimetic**: BV6 (D)	**Targets**: cIAP1, cIAP2, XIAP	IFNα TRAIL TNFα	• BV6, LCL161 and Birinipant in combination with IFNα synergistically induce GBM cell apoptosis via DISC formation • IFNα and BV6 increase TNFα and TRAIl production by GBM cells. TNFα not required for A172-induced cell death. • TRAIL-DR5 signaling responsible for noted GBM cell death. • A172 cells resistant to BV6 + TNFα. Susceptible to TRAIL-induced apoptosis, alone and in combination with BV6	[132]
	Birinapant (D)				
	LCL161 (M)				
SNB75 (H, Imm)	**Smac Mimetic**: LCL161 (M)	**Targets**: cIAP1, cIAP2, XIAP	VSVΔ51	• LCL161 significantly reduces SNB75 GBM cell viability in combination with innate immune stimuli including oncolytic viruses	[133]
Primary GSCs: GBM6 (H, P) GBM9 (H, P) GBM40 (H, P) Non-GSC enriched: RNS175 (H, P)	**Smac Mimetic**: GDC-0152 (M)	**Targets**: cIAP1, cIAP2, XIAP, ML-IAP		• Repeat treatment of 10-fold dilution series of GDC-0152 (10µM– 0.01nM) over 8 days did not affect GBM9 GSC-cell viability in normoxia, significantly reduced in hypoxia • GDC-0152 treatment in normoxia reduced stem-cell characteristics of GSC lines. In hypoxia, GDC-0152 significantly increased cleaved caspase-3 levels, TNFα expression and reduced overall viability	[134]
A172 (H, Imm) T98G (H, Imm)	**Smac Mimetic**: BV6 (D)	**Targets**: cIAP1, cIAP2, XIAP	TMZ	• BV6 and TMZ induce significant apoptosis of GBM cells • BV6 increased IFNβ production by GBM cells. IFNβ in combination with TMZ significantly increases GBM cell apoptosis. IFNβ is required for BV6-TMZ synergism	[135]
A172 (H, Imm) U87MG (H, Imm)	**Smac Mimetic**: BV6 (D)	**Targets**: cIAP1, cIAP2, XIAP	TMZ	• BV6 and TMZ treatment induces reactive oxygen species production by GBM cells prior to induction of apoptosis • ROS production contributes to BAX activation and subsequent apoptosis	[136]
U87MG (H, Imm) T98G (H, Imm) A172 (H, Imm) U118MG (H, Imm) U138MG (H, Imm) GBM1, GBM2, GBM3 (H, P) GBM10 (H, P, GSC enriched)	**Smac Mimetic**: BV6 (D)	**Targets**: cIAP1, cIAP2, XIAP	Drozitumab (TRAILR2 engaging antibody)	• TRAILR2 expressed at significantly higher levels than TRAILR1 in all tested human GBM cells • BV6-Drozitumab cotreatment induces significant cell death in all tested GBM cells in vitro via DISC formation requiring RIP1. No single agent effects • BV6-Drozitumab reduces tumor size in vivo, colony formation in vitro • In A172 and U87MG cells, caspase-8 activation, Bid cleavage to tBid and subsequent caspase-9 and caspase-3 cleavage and activation are seen only following Dorzitumab treatment. Maximal Bid cleavage (and caspase-9 activation) following addition of BV6 • cFLIP is major resistance factor. BV6 increased cFLIP expression. BV6-Drozitumab cotreatment reduced cFLIP levels • TNFα not involved in cotreatment efficacy	[137]
U87MG (H, Imm) LN18 (H, Imm) LN229 (H, Imm) SMA560 (M, Imm)	**Smac Mimetic**: Smac peptides containing AVPIAQK N-terminal amino acids of endogenous Smac		TRAIL αCD95 antibody (apo1) Doxorubicin	• Smac overexpression sensitizes GBM cells to TRAIL, CD95 engagement and doxorubicin, even when GBM cells overexpress BCL-2 • TRAIL alone induces significant apoptosis only in GBM cells (murine and human). No effect on astrocytes, Schwann cells, or other normal tissue. Enhanced by Smac cotreatment • In vivo, Smac + TRAIL treatment induced significant U87MG apoptosis, reduced tumor growth and extended mouse survival with long-term cures. Smac alone had no impact.	[138]
A172 (H, Imm)	**Smac Mimetic**: BV6 (D)	**Targets**: cIAP1, cIAP2, XIAP		• TNFα-TNFR1 signaling not required for BV6-mediated apoptosis of A172 cells • NF-κB required for BV6-mediated apoptosis of A172 cells (canonical and non-canonical) • BV6 increases DR5 expression, in turn required for apoptotic effects • Soluble TRAIL minimally contributes to observed apoptosis in vitro	[139]
U87MG (H, Imm) GSCs (H, P)	**Smac Mimetic**: AZD5582 (D) SM-164 (D) encapsulated in liposomes grafted with rabies virus glycoprotein and lactoferrin	**Targets**: cIAP1, cIAP2, XIAP		• Liposome encasing improved BBB crossing capacity of Smac mimetics • Both tested Smac mimetics induced significant apoptosis of tested GBM cells	[140]
M059K (H, Imm) SNB75 (H, Imm) U118 (H, Imm) CT2A (M, Imm) GL261 (M, Imm) BT30, BT48, BT69 (GSCs, H, P)	**Smac Mimetic**: LCL161 (M)	**Targets**: cIAP1, cIAP2, XIAP	VSVΔ51 TNFα	• Mouse and tested human GBM cells sensitive to LCL161 and TNFα cotreatment • LCL161 combination with PD-1 blockade induces durable cures in murine GBM models	[141]

*H* Human, *M* Mouse, *Imm* Immortalized, *P* Primary, *D* Dimeric, *M* Monomeric, *I* Intrinsic Pathway, *E* Extrinsic pathway, *CC3* cleaved Caspase-3

**Table 2:** Direct apoptotic pathway targeting agents in GBM clinical trials

Identifier	GBM type	Drug name	Target & pathway	Phase	Results
NCT00390403	Newly diagnosed GBM	AT-101/R-(-)-Gossypol	BCL-2 BCL-xL (Intrinsic)	I	Not posted
NCT00540722	Progressive or recurrent GBM	AT-101/R-(-)-Gossypol	BCL-2 BCL-xL (Intrinsic)	II	CR: 0%
					PR: 1.8%
					SD: 26.8%
					P: 62.5%
					PFS: 1.87 months
NCT03020017	Glioblastoma or Gliosarcoma treated with surgery	NU-0129 encapsulated in gold nanoparticles bearing spherical nucleic acids on the surface	*BCL2L12* gene (Intrinsic)	0	No adverse events related to drug
					Drug successfully accumulated in tumor tissue following intravenous administration
NCT04573192	Glioblastoma at first progression	L19TNF (TNF-α attached to scFv fragment of L19 antibody specific for fibronectin)	TNF-α (Extrinsic)	I/II	Ongoing

*CR* Complete response, *PR* Partial response, *SD* stable disease, *P* progression, *OS* overall survival, *MS* Median Survival, *PFS* progression free survival

### Intrinsic apoptotic regulation in the CNS

#### Neural stem/progenitor cell sensitivity

Given the diversity of cell types and their associated roles within the CNS, it is unsurprising that significant differences in the regulation of cell death exist between populations. NSCs and NPCs (herein used interchangeably) are primarily located in the SVZ of the lateral ventricles and the subgranular zone of the hippocampus. Furthermore, NSCs/NPCs are capable of self-renewal, migration (primarily to the olfactory bulb and to injured regions), and differentiation into neural or glial progeny [142–146]. NSCs are particularly enriched at sites of neural injury, able to survive exposure to inflammatory signals and death ligands [142], which makes them particularly attractive therapeutic targets in the treatment of traumatic brain injury, stroke and neurodegenerative conditions [147, 148]. NSC survival and proliferation are dependent on PI3K-AKT and MAPK signaling, initiated via access to fibroblast growth factor 2 (FGF), epidermal growth factor (EGF) and insulin-like growth factor 1 (IGF1) [149]. During development, NSCs and NPCs die primarily through the intrinsic apoptotic pathway [150–154] with high sensitivity to growth factor withdrawal, changes in calcium ion flux [155] and ischemia/hypoxia [156]. Deficiency in components of the intrinsic pathway results in fatal over accumulation of NPCs, although caspase-independent mechanisms of cell death occur at low levels [157].

#### Neuron sensitivity

More neurons are produced during development than are necessary. The excess cells are primarily removed by apoptosis to optimize neuronal network connectivity. Once fully integrated into networks, neurons become post-mitotic and typically persist throughout the lifespan of an organism. Given this lack of capacity for replication, minimal neurogenesis from NSC pools, and consequent requirement for a long lifespan, it follows that as neurons mature, their sensitivity to intrinsic apoptotic cues such as growth factor withdrawal substantially decreases [158]. An exception is oxidative stress. High oxygen consumption and minimal antioxidant capacity necessitates a reliance on astrocytes for combatting ROS damage via release of glutathione, ascorbate and superoxide dismutases [159–161]. This relationship is significantly perturbed and exacerbates neuronal cell death in the context of neurodegenerative conditions. Multiple restrictions to the c-Jun N-terminal kinase (JNK) pathway further limit intrinsic apoptosis. The JNK pathway is activated following exposure to numerous cell death stimuli, upregulating pro-apoptotic BCL-2 and BH3-only family members [162]. Further regulation is provided through developmental changes in BCL-2 family protein expression. Unlike NPCs, which express high levels of both BAX and BAK [163], mature neurons express low levels of BAX (which requires translocation to the mitochondria and oligomerization) but no BAK (which is always present in the mitochondrial membrane), potentially representing an opportunity for increased anti-apoptotic BCL-2 family regulation [164–174]. BCL-xL [175, 176], BCL-2 [177], and BCL-w [178, 179] are highly expressed, while pro-apoptotic BH3-only protein expression is significantly reduced relative to NPCs. These proteins are inducible, however the apoptotic stimuli must be strong enough to overcome the heavily anti-apoptotic skewing [162].

Near complete transcriptional repression of APAF-1 further limits neuronal apoptosis through the intrinsic pathway [180–186]. CYCS is kept in its reduced, inactivated state [187] and low expression levels of CASP3 and CASP7 in mature neurons further limits apoptotic sensitivity [182, 188]. Nerve growth factor (NGF) is necessary for mature neuron survival, reducing active CASP3 levels via lysosomal degradation, inhibiting CYCS loss from the mitochondria via PI3K alterations, and regulating BH3-only protein expression [189, 190]. NGF withdrawal consequently increases CASP3 activation [191]. Global NGF withdrawal leads to cell death, while local deficits lead to synaptic pruning [192]. More important than growth factors in promoting neuronal survival is neurotransmitter input, acting to increase expression of anti-apoptotic proteins such as BCL-2 and reducing BAX and CASP9 [193–196]. Synaptic activity affects the expression of Brain Derived Neurotrophic Factor (BDNF) and Glial Cell Derived Neurotrophic Factor (GDNF), and these two factors further promote neuroprotection similar to NGF as described above [197, 198]. Kole et al. suggest that neuronal death seen in the context of neurodegenerative conditions may be a result of a reversion to a more immature, death-sensitive phenotype resembling NPCs [158].

#### Astrocyte sensitivity

Astrocytes are the most numerous cells in the brain, with a myriad of roles in maintaining CNS homeostasis including neuroimmune regulation, blood brain barrier (BBB) maintenance, neurotransmitter clearance, angiogenesis, and promoting neuron signaling and survival through metabolic and trophic support. Lactate from astrocytes is a major neuronal energy source. Plasticity of astrocyte reactive states, triggered by CNS injury, inflammation, ROS, and cell stress signals, allows for rapid responses characterized by migration to the injury site, inflammatory cytokine, chemokine and ROS release, hypertrophy, proliferation (termed astrogliosis), and, depending on the extent of injury, formation of the glial scar. This scar is protective over the short term but ultimately prevents CNS regeneration through the injury site over the long term. BBB regeneration, control of blood flow and antioxidant action through glutathione release are key astrocyte roles following CNS injury [199].

Reactive astrogliosis is followed by significant astrocyte cell death [200]. The release of cytokines and ROS can induce further neurodegeneration if the reaction is prolonged [201], resulting in BAX mitochondrial translocation and initiation of the intrinsic apoptosis in both astrocytes and neurons [202]. As in NSCs/NPCs, astrocytes are sensitive to insults activating the mitochondrial apoptotic cascade, including excess intracellular calcium ions, oxidative stress, ischemia and UV radiation (reviewed in [203]). Neurotrophic factors (BDNF, GDNF, bFGF, IGF1, EGF, NGF) protect against induction of the intrinsic apoptotic cascade via tropomyosin-related receptor kinase (TRK) receptor signaling through PI3K-AKT and MAPK pathways [204, 205]. The brain is especially susceptible to oxidative stress given high oxygen demands, high levels of redox transition metal ions and low antioxidant enzymes [206]. Oxidative DNA damaging agents, such as cytosine arabinoside, are especially effective in inducing apoptosis [207]. 14-3-3γ, one of seven 14-3-3 protein isotypes, is most highly expressed in the brain. Its expression is significantly increased in astrocytes following ischemia as a result of JNK pathway activation. Binding of 14-3-3γ to BAD prevents induction of mitochondrial apoptosis, and in vitro promotes astrocyte survival under ischemic conditions. In neurons, 14-3-3γ expression is regulated by ERK1/2 and MAPK, potentially resulting in differences between the two cell types in sensitivity to ischemia [208].

#### Oligodendrocyte sensitivity

Oligodendrocytes are postmitotic glia appearing after neurons and astrocytes during neurodevelopment. Responsible for the formation and maintenance of myelin sheaths on neuronal axons, oligodendrocytes are generated in excess, undergoing significant cell death to better match the number of axons [209]. Each oligodendrocyte can myelinate multiple axons and these cells are critical for neuron saltatory conduction and axonal cytoskeleton integrity. Oligodendrocytes further act as a source of neuronal energy and trophic factors, in turn receiving critical trophic support from axons [210, 211]. They develop from immature OPCs following several direct differentiation steps in response to reductions in mature oligodendrocytes, from OPCs (A2B5^+^NG2^+^O4^−^) to pre/pro-oligodendrocytes (late OPCs; A2B5^+^O4^+^O1^−^) to immature oligodendrocytes to mature oligodendrocytes (reviewed in [212]) [213–216], each with differing sensitivities to both intrinsic and extrinsic apoptotic cues.

OPCs are the primary dividing cell in the CNS, with an extended G1 phase [217, 218]. Evidence points to OPCs as a major cell of origin for oligodendroglioma and other malignant gliomas [219–221]. In the adult CNS, OPCs extend motile filopodia to survey the surrounding environment, move continuously throughout the cortex and are found uniformly between cortical layers. Loss of OPCs through cell death or differentiation results in rapid proliferation and migration to maintain distribution, contributing to a near constant turnover [222]. With few exceptions, cells of the oligodendrocyte lineage are very sensitive to mitochondrial cell death. Relative resistance to intrinsic apoptotic death stimuli increases as differentiation progresses. For example, inhibitors of heat shock protein 90 (HSP90, a stress chaperone commonly used as a BBB permeable chemotherapeutic tool) effectively kill OPCs at nanomolar concentrations, while pre-oligodendrocytes require nearly a thousandfold higher dose to effect similar cell death response [223]. OPCs are sensitive to X-ray irradiation, with substantial, rapid induction of apoptosis following exposure to doses above 10 Gy [224]. Levels of PARP are significantly higher in fetal OPCs relative to adult, consistent with lower DNA repair capacity with aging. Fetal OPCs are significantly more sensitive to the cytotoxic effects of PARP inhibition, further acting to limit proliferation and differentiation [225]. Fetal OPCs and pre-oligodendrocytes are more susceptible to oxygen and glucose deprivation than mature oligodendrocytes or their adult counterparts and are notably more sensitive to inflammation and said deprivation relative to astrocytes. This is due to the dependence of fetal OPCs on glycolysis for survival and differentiation; hypoglycemia prevents OPC maturation and triggers cell death [226].

Among all cells in the CNS, oligodendrocytes have the highest metabolism which, coupled with low glutathione and consequent poor antioxidant capacity, make these cells highly susceptible to metabolic stress [227–229]. Mature oligodendrocytes are substantially more resistant to oxidative stress as a result of significantly higher glutathione levels [230–232]. Signaling through PI3K is required for oligodendrocyte and OPC survival, regardless of the presence of growth factors [233]. Osteopontin, a glycosylated phosphoprotein, protects OPCs from oxidative stress-induced cell death upon hydrogen peroxide exposure by limiting the characteristic BAX and BID increases and BCL-2 and BCL-xL decreases [234]. The pro-death mediator Cytoplasmic BCL-2 nineteen kilodalton interacting protein 3 (BNIP3) is significantly increased following oxygen-glucose deprivation, and acts to mediate OPC cell death following ischemia [235]. Nerve-glial antigen 2 (NG2), a characteristic marker of OPCs, is a proteoglycan involved in OPC migration [236] and has also been found to sequester HTRA2, therein reducing OPC sensitivity to oxidative stress [237]. Lack of BDNF contributes to reductions in OPC proliferation and, consequently, remyelination efforts in the context of demyelinating injury [238]. Oligodendrocytes of multiple developmental stages are highly sensitive to excitotoxicity [239]. This excitotoxicity is dependent on increased BAX association with BID or BAD and reduced interactions with BCL-xL [240]. OPCs are more sensitive to excitotoxic necrosis compared to mature oligodendrocytes, but express more TRKC (for NT3 ligand) receptors [241], illustrating an increased growth factor requirement for survival. Increased sensitivity relative to mature oligodendrocytes is due to a high ratio of BAX to BCL-xL (OPCs express higher BAX), as well as higher pro-CASP3 expression. Mature oligodendrocytes have a lower BAX: BCL-xL ratio [242, 243]. Caspase inhibition only partially blocks these effects [242], suggesting concurrent activation of alternative forms of cell death.

#### Summation

The near-complete shutdown of the intrinsic cascade in GBM is strikingly similar to the pattern seen in neurons, although without the sensitivity to excitotoxicity and ROS given high glutamate efflux transporter and antioxidant expression [244–247]. The heavy anti-apoptotic skewing of BCL-2 family members, variable pro-CASP3 levels, low APAF-1 and pro-CASP9, and high XIAP and Survivin suggest targeting the intrinsic cascade alone is likely to only be marginally effective at tolerable treatment doses. It will likely prove advantageous to target the extrinsic pathway in combination, especially in GBMs expressing BID.

## Regulation of cell death through the extrinsic apoptotic pathway

### Extrinsic apoptotic regulation in the CNS

#### Neural stem/progenitor cells sensitivity

While fatal overaccumulation of NSCs occurs in the context of defects in intrinsic apoptotic pathway members, cells develop normally in mice lacking CASP8 [248]. Ricci-Vitiani et al. looked at components of the extrinsic apoptotic cascade in primary human NPCs. They found that both embryonic and adult NPCs were completely resistant to FASL, TRAIL and TNF-α induced cell death despite these cells expressing high levels of FAS and DR5 [249, 250]. Human NSCs express low levels of TNFR1. Sensitivity to TNF-α can be induced with cycloheximide, which blocks protein synthesis, and cell death occurs through apoptosis and, to a slightly lesser extent, necroptosis [251]. While FADD and CASP3 are expressed, human NPCs showed a near complete lack of CASP8 [249]. CASP8 expression could be induced via exposure to multiple inflammatory cytokines (such as TNF-α, IFN-γ, IL-1β). However, resistance to FASL and TRAIL is maintained through significant upregulation of PEA-15. NPCs express little cFLIP [249] and it has no role in NSC/NPC response to death ligands [252].

The immortalized embryonic mouse NSC/NPC cell line C17.2 express FAS with low levels of pro-CASP8 relative to Jurkat T-cells. No activation of CASP3 or CASP8 are seen upon FAS agonism. In agreement with Ricci-Vitiani et al., C17.2 resistance to FAS engagement was not due to cFLIP. ERK activation was increased following treatment with a FAS agonist [253], which, though unexplored in that study, may be due to the overlapping actions of PEA-15 [72]. Exposure of NSCs to FAS results in activation of ERK, which promotes proliferation [252]. Primary early postnatal mouse NPCs express high levels of FAS and low FASL. Strikingly, Knight et al. found that mice NPCs express FAS at levels similar to T-cells, and treatment with exogenous FASL reduced spontaneous apoptosis in vitro, illustrating a protective function of the FASL-FAS pathway in NSCs. Indeed, while NPCs are highly sensitive to EGF and FGF withdrawal, treatment with exogenous FASL reduces such cell death without altering proliferation rates or differentiation [254]. Corsini et al. found that FAS signaling enhanced NPC survival and neuronal differentiation through PI3K and protects against cell death in global ischemic injury models, with roles in working memory formation [255]. In examining anti-apoptotic gene and protein expression, mouse NPCs were found to express little cFLIP or CASP8. Upon FASL exposure, no changes in cFLIP activation were noted, and CASP8 and -3 remained steady and inactive [254].

Conflicting findings regarding death ligand sensitivity have been reported. For example, Ivanov and Hei found that human NSCs were dose-dependently susceptible to γ-radiation induced mitochondrial apoptosis. They also showed NSCs expressed pro-CASP8 at a basal level, showed decreased Survivin upon irradiation, and upregulated TRAIL expression, which then induced autocrine and paracrine extrinsic apoptotic signaling. Human NSCs were also susceptible to exogenous TRAIL-induced apoptosis. cFLIP levels did not change regardless of radiation dose or TRAIL exposure [256, 257]. Both murine [258] and human NSCs [256, 257] show p53-mediated apoptosis in response to increasing doses of radiation. Consistent with FAS possessing a p53 response element, NPCs were found to be sensitive to FAS-induced apoptosis upon exposure to radiation. Radiation increased FAS expression on the plasma membrane with increased sensitivity to FAS agonism [258]. Inhibition of PARP1 in mouse embryonic NSCs/NPCs induces apoptosis through increased p53 activity, with the expression of p21 [259–261], NOXA, PUMA, TRAIL-R2/DR5 and FAS increasing prior to pro-CASP8 cleavage and activation [262], suggesting functional extrinsic apoptotic signaling. However, Fukuda et al. found that while overexpression of XIAP prevents CASP9 and CASP3 activation in rat NSCs/NPCs, this did not rescue cell death in response to radiation. Rather, the cells shifted from caspase-dependent to caspase-independent modes, likely via AIF translocation to the nucleus [263]. Reducing AIF protects these cells by inhibiting cell death and proliferation [264].

#### Neuron sensitivity

While sensitivity to intrinsic apoptotic triggers is drastically reduced during differentiation and maturation, neurons conversely become more susceptible to death ligands. In comparing NPCs to neurons, Ricci-Vitiani et al. found significant increases in CASP8 expression associated with neuronal differentiation and opposite responses to inflammatory factors. TNF-α, IFN-γ and IL-1β all increase neuron CASP8 and FAS expression. Combination treatment with all three sensitizes neurons to substantial FASL-induced cell death, whereas NPCs were completely resistant. Neurons express low FAS and are insensitive to FASL-induced cell death without inflammatory induction of CASP8 [249]. Along with inflammation, ischemia also sensitizes neurons to death ligands. TRAIL, FASL and c-Jun are induced in neurons following ischemic injury, while TNF-α levels fluctuate. Indeed, TRAIL-induced apoptosis represents a major component of neuronal cell death in response to ischemia [265], as well as in neurodegenerative conditions such as Alzheimer’s disease (AD) [266, 267].

FASL is expressed at extremely low levels in the CNS, which (along with high levels of FAS) acts to limit neuroinflammation [268]. This is important given the sensitivity of neurons to death ligands; regulation of FAS-FASL signaling is disrupted in numerous neurodegenerative conditions [269–272]. Instead, under homeostatic conditions, FAS-FASL signaling is used to mediate neurite outgrowth through MAPK, ERK and NF-κB signaling [273, 274]. Astrocytes protect against FAS-mediated extrinsic apoptosis in neurons following exposure to radiation, releasing BDNF and GDNF to reduce FAS expression and limit CASP8 and FADD expression and activation [275]. PEA-15 is expressed at lower levels than NPCs, and unlike NPCs inflammation and injury decreases PEA-15 expression in neurons [249]. Nonetheless, under homeostatic conditions PEA-15 is widely expressed in neurons throughout the brain [73]. Neurons transduced with PEA-15 resist ROS-induced cell death, increase BCL-2 expression, and further promote survival via alterations in AKT, ERK and JNK phosphorylation [276]. Neuronal PEA-15 levels are also altered in psychiatric conditions, including major depression and schizophrenia [277]. cFLIP plays a relatively larger role in apoptosis prevention compared to NSCs, notably during hypoxia, ischemic injury, growth factor withdrawal, spinal cord injury and glucose deprivation, primarily through promotion of NF-κB signaling [278–280]. However, its role in extrinsic apoptosis prevention is likely still minimal, as it has no role in neuronal resistance to FASL [281]. The necroptotic pathway is fully functional in neurons, with RIPK1 and RIPK3 expression, and subsequent interactions with MLKL, significantly increased following hypoxia-ischemia [282].

The long isoform of FAS apoptotic inhibitory molecule (FAIM-L), a splice variant of FAIM1, is exclusively expressed in neurons, is induced by NGF, is activated primarily through MAPK/ERK, PI3K/AKT and NF-κB signaling, and protects against both FAS and TNF-α induced cell death [283]. Expression of FAIM-L is increased in correlation with levels of neuronal death receptors [273, 284–286]. FAIM-L directly binds XIAP via IAP-binding motif interactions at the BIR2 domain, preventing its autoubiquitination and proteasomal degradation. FAIM-L protects type 2, but not type 1, cells from apoptosis. Without FAIM-L, neuronal XIAP levels are dramatically reduced [287] and the neuronal cells become sensitive to TNF-α and FAS-induced apoptosis. However, modifications to the expression of DISC and DIC2 inhibitors following spinal cord injury shift neurons into type 1 cell death [280]. FAIM-L XIAP stabilization implicates FAIM-L in a myriad of neuronal processes, including synaptic plasticity and long term depression, regulation of glutamatergic receptor subunit internalization, and inhibition of axon degeneration [288]. Further, FAIM-L directly competes with FADD for interactions with death receptors, preventing DISC and DIC2 formation [283, 289]. As such, FAIM-L represents a dual functioning, neuron-specific anti-apoptotic protein, protecting XIAP from degradation and preventing downstream extrinsic apoptotic signaling.

Contrary to its name as tumor necrosis factor alpha, TNF-α itself is not lethal to neurons. TNF-α is constitutively expressed at low levels in the CNS (along with TNFR1 and TNFR2) and plays a neuroprotective role. Along with cIAP1 and XIAP, FAIM-L is also critical for the neuroprotective function of TNF-α, and loss of FAIM-L expression is an early event contributing to AD neurodegeneration [290, 291]. Lack of TNFR1 and TNFR2 exacerbates cerebral ischemia-induced apoptosis in mouse neurons [292] and TNF-α pre-treatment protects neurons from numerous toxic insults and disease states through increased NF-κB signaling [293–295]. In the absence of functional CASP8, the necroptotic machinery is present in neurons [296, 297]. Astrocytes affected by amyotrophic lateral sclerosis induce necroptosis in motor neurons [298].

Neurons express TRAILR1 and TRAILR2 and are sensitive to TRAIL-induced apoptosis. TRAIL is not expressed in the CNS under homeostatic conditions [299–304], consistent with the relatively minimal peripheral immune populations present within the CNS milieu. Inflammatory cytokines TNF-α and IFN-γ significantly increase neuronal TRAILR2 expression and TRAIL expression within the CNS, as do ischemia, glucose deprivation and neuropathologies [303, 305]. Similar upregulations are found in neurons of multiple sclerosis patients [306], and pro-inflammatory cytokine signaling through Glucocorticoid-Induced TNFR-Related (GITR)-GITRL potentiates TRAIL-induced neuronal cell death [307]. Like FAS-FASL, TRAIL signaling significantly contributes to ischemic neuronal cell death [265]. Ischemia induces increased TRAIL expression by astrocytes and microglia with a concomitant increase of TRAILR2 expression in neurons, contributing to neurodegeneration; TRAILR2 blockade is neuroprotective in ischemic stroke models [308]. TRAIL signaling plays similar dual roles as TNF-TNFR in both potentiating EAE/MS pathology while limiting neuroinflammation through induction of immune cell death [309, 310]. West Nile virus (WNV) increases the infiltration of CD8^+^ T-cells into the brain, which express TRAIL and cause neuronal cell death. Further, WNV increases TNF-α and FAS expression, with concomitant increases in neuronal cIAP2, cFLIP, TNFR1, TNFR2 and CASP8 [311]. Neuronal death in the context of HIV-encephalopathy is via TRAIL from HIV-infected monocytes [302, 312], and infiltrating macrophages expressing TRAIL contribute to several neurodegenerative conditions [305].

#### Astrocyte sensitivity

Given their role in inflammation and responses to injury, astrocytes are generally resistant to extrinsic apoptotic triggers. The question of whether astrocytes express CASP8 has generated conflicting results. Yew et al. found no CASP8 protein expression by astrocytes in the brains of normal or AD patient [313] and Wosik et al. found similar lack of CASP8 in fetal human astrocytes [314]. Barca et al. found that while fetal rat astrocytes express abundant CASP8 and are sensitive to FAS/FASL-induced cell death, neonatal astrocytes showed transcriptional inactivation of the CASP8 gene with undetectable mRNA levels and resistance to FAS-induced apoptosis [315]. More recent studies have disputed this, showing basal expression of CASP8 in human astrocytes that is further induced upon death receptor stimulation [78, 316], an induction which is also noted in fetal astrocytes [314]. Thus, CASP8 expression in astrocytes appears to be developmentally regulated, substantially decreasing with age but its expression is inducible.

Astrocytes express high levels of FAS and FASL, with astrocyte FASL functioning to induce apoptosis of invading activated T-cells [309, 317, 318], a mechanism used in numerous immune privileged organs to resolve inflammation [319–321]. Indeed, neurons are susceptible to cell death induced by T-cells regardless of antigen specificity, likely a result of FAS-FASL and TRAIL-TRAILR1/R2 interactions. Astrocytes resist this killing and, in turn, induce T-cell apoptosis as a protective mechanism [322]. At a basal level, astrocytes express minimal FASL and do not undergo apoptosis upon engagement of FAS [323] or TNFR1. Resistance to FAS-FASL is due to high levels of phosphorylated, activated CAMKII, which increases the expression and phosphorylation of PEA-15 and cFLIP [78], illustrating that astrocytes use two complementary, overlapping mechanisms of DISC inhibition. Similar increases of cFLIP are seen in fetal astrocytes upon exposure to death ligands and inflammatory cytokines [314]. In human astrocytes, FAS-FASL primarily triggers IL-8 production and CXCR2 expression, and through autocrine and paracrine signaling this acts to prevent apoptosis [324] likely through PI3K-AKT, NF-κB, AP-1, STAT3 and/or β-Catenin signaling (reviewed in [325]). Inflammatory cytokines increase astrocyte FAS/FASL expression [326, 327] concurrent with induction of reactive states [328–330]. Exposure to IFN-γ leads to increased TNFR1 and FAS expression by human astrocytes, but only sensitizes them to FAS-FASL cell death [331]. Astrocyte reactivity affects sensitivity to FAS, but not TNF-α and TRAIL-mediated cell death. Resting astrocytes resist all extrinsic apoptotic triggers, while reactive astrocytes, primed by strong enough inflammatory cues become sensitive to FAS-FASL. The sensitivity of reactive astrocytes is mediated via increases in CASP8 expression and further upregulation of FAS with no significant cFLIP expression changes and followed by FADD recruitment and DISC formation [328]. Consistent with the idea that astrocytes are type 1 cells, FASL-induced apoptosis of reactive astrocytes following infection with Ectromelia virus involved activation of CASP8 and − 3 with no involvement of CASP9 [332]. Further, myricetin, a flavonol with anti-viral, anti-oxidant, anti-inflammatory and proapoptotic functions, reduces cFLIP and BCL-2, but shows no effect on astrocyte TRAIL sensitivity [333].

Levels of TRAILR1 and TRAILR2 are too low for sufficient TRAIL binding to induce DISC formation [78], a feature also found in fetal astrocytes [334], and can be the major mechanism of astrocyte resistance to TRAIL. Astrocytes express TRAIL decoy receptors (TRAILR3 and TRAILR4), which have no DD and sequester ligand from TRAILR1/R2 [299, 335–337]. This lack of TRAILR2 expression is very stable. Treatment with arsenic trioxide, which upregulates TRAILR2 through CCAAT enhancer binding protein homologous protein (CHOP; also absent or expressed at low levels in astrocytes), showed no such effect on human astrocytes, which remained resistant to TRAIL [338] regardless of source or delivery method [339]. BNIP3 is a pro-cell death BCL-2 family member capable of opening the mitochondrial permeability transition pore and inducing caspase-independent cell death when in the cytoplasm. Nuclear localization leads to binding of BNIP3 to the TRAILR2 promoter, repressing TRAILR2 expression, and thereby inhibiting TRAIL-induced apoptosis [340]. Human astrocytes show substantial nuclear localization of BNIP3 [341], which may partially account for the stable, near complete absence of TRAILR2 expression. While normal human astrocytes resist soluble and synthesized crosslinked TRAIL, combination with chemotherapeutics including cisplatin caused substantial apoptosis [342], consistent with the aforementioned sensitivity to mitochondrial apoptotic cascade initiators. Consistent with the ability of ROS to trigger astrogliosis, as well as the subsequent apoptosis of excess astrocytes, hydrogen peroxide is capable of inducing significant increases in TRAILR2 expression in human astrocytes, leading to significant apoptosis upon challenge with TRAIL [343].

Pinoresinol, a lignin from *Rubia philippinensis*, has recently been shown to inhibit the long isoform of cFLIP, which sensitizes cells to TRAIL-induced apoptosis. Despite significant cFLIP reductions, astrocytes remained resistant to TRAIL [344, 345], likely due to both inadequate TRAILR1/R2 expression and the redundant actions of PEA-15. TRAIL is expressed at very low levels in the CNS under homeostatic conditions– only reactive astrocytes show expression [346, 347].

#### Oligodendrocyte sensitivity

OPCs and pro-oligodendrocytes are sensitive to TNF-α-induced cell death, representing the primary targets during infant white matter injury, while immature and mature oligodendrocytes are more resistant, despite little developmental change in TNFR1 expression [348–350]. OPCs show significant upregulation of members of the extrinsic apoptotic cascade relative to differentiated oligodendrocytes, with significantly higher levels of CD40-CD154, CD27, FASL, 4-1BB, TRAIL and DR3. CASP8 and CASP10, as well as FAS and TRAILR1, also showed consistently higher expression compared to in their more differentiated counterparts. Greater expression of death receptor cascade proteins corresponded to the enhanced susceptibility to death of OPCs to TNF-α, FAS and TRAIL [351]. Proliferating OPCs in particular undergo significant apoptosis in response to IFN-γ, signaling via STAT and MEK-ERK pathways [352]. TNF-α substantially increases cell death by IFN-γ [353, 354], likely due to IFN-γ induced TNFR upregulation [355]. At low doses, both IFN-γ and TNF-α prevent OPC cell cycle exit and differentiation, improving survival and leading to accumulation. At high dose, apoptosis occurs [356].

Significant neonatal OPC death is seen following increase of TNF-α in the context of periventricular leukomalacia, a form of brain injury most commonly seen in premature babies. This cytotoxic effect is prevented by treatment with IGF-1 signaling through the PI3K-AKT pathway, resulting in reduced BAX mitochondrial localization, BAD phosphorylation, and CASP9 and CASP3 inhibition [357]. BAX deletion also prevents TNF-α-induced cell death [358], as do corticosteroids [354]. These protective effects through alterations of members of the intrinsic apoptotic cascade suggest oligodendrocyte lineage cells possess high XIAP-to-caspase ratios and are type 2. Signaling through TNFR2 is beneficial to OPC proliferation, differentiation and remyelination [359], while levels of TNF-α associated with EAE and other inflammatory conditions exacerbate demyelination [360, 361] through TNFR1, a duality behind the worsening of disease upon treatment via indiscriminate TNF blockade [362, 363]. TNFR2 signaling is crucial for OPC survival and differentiation during oxidative stress and MS [359, 364–367].

Similar to the case with TNF-α, human oligodendrocyte development is accompanied by a reduction in sensitivity to TRAIL due to shifts from TRAILR1 and TRAILR2 being dominantly expressed in OPCs and pre-oligodendrocytes whereas the decoy receptors TRAILR3 and TRAILR4 most highly expressed in immature and mature oligodendrocytes [368]. Nonetheless, human oligodendrocytes express all four TRAIL receptors and are sensitive to TRAIL-induced apoptosis in vitro [369, 370], and TRAIL likely plays a role in oligodendrocyte cell death in MS [371]. OPCs upregulate TRAILR2 expression following hypoxia-ischemia. Primary OPCs are not sensitive to TRAIL but concurrent oxygen-glucose deprivation and/or exposure to TNF-α and IFN-γ significantly increases sensitivity [303]. p53 expression can increase TRAILR1, TRAILR2, and FAS, increasing sensitivity to death ligands [372]. Cortical white matter lesions in multiple sclerosis patients display reduced CASP8 cleavage and activation coinciding with increases in cFLIP expression [373], in keeping with oligodendrocyte lineage cells being the major cFLIP expressing populations in the CNS along with microglia [374].

Human oligodendrocytes are sensitive to FAS-FASL induced apoptosis in vitro [375, 376]. Hyperoxia causes increased FAS expression on OPCs, with consequent CASP8 and CASP3 activation and apoptosis [377]. Necroptosis is a common OPC response following hypoxia-ischemia. OPCs express high levels of RIPK3, which interacts with RIPK3, RIPK1, MLKL and/or CAMKIIδ. Complexes between RIPK3 and MLKL or CAMKIIδ are required for OPC necroptosis to proceed. CAMKIIδ phosphorylation by RIPK3 leads to opening of the mitochondrial permeability transition pore, while MLKL tetramers disrupt OPC membrane integrity. Inhibition of either RIPK3 interaction results in significantly reduced OPC death under hypoxic-ischemic conditions [378].

#### Summation

In NSCs/NPCs of multiple species, the mitochondrial apoptotic pathway is intact whereas the extrinsic pathway is inactive (through low CASP8 expression and high and readily inducible levels of active PEA-15). Neuronal differentiation from NSCs is associated with a reversal in sensitivities. While NSCs potently resist death ligands and the apoptotic effects of inflammatory cytokines, neurons are tremendously sensitive, likely explaining the extensive immune exclusion from the CNS. Conversely, NSCs die predominantly through the mitochondrial apoptotic cascade during development, while neurons show a near complete shutdown of this pathway via differential expression of numerous cell death proteins which are severely altered in the context of injury, aging, and neurodegenerative conditions. Regulation of cell death in astrocytes appears similar to that seen in NSCs. Sensitivity to inducers of the intrinsic apoptotic cascade is coupled with multifaceted resistance to death ligands, including low CASP8 expression that decreases with age, multiple inhibitors of DISC formation, and a stable, near complete shutdown of TRAILR2 expression. Plasticity of astrocyte reactive states allows for sensitization to initiators of the extrinsic apoptotic pathway as a means of terminating inflammatory and proliferative reactions to injury, pathology or infection. Oligodendrocytes are one of the most energetically demanding and high turnover cell populations in the CNS and are incredibly sensitive to metabolic and oxidative stress. Sensitivity to cell death cues triggering either apoptotic pathway decreases as OPCs differentiate, with maturation and aging accompanied by further characteristic changes. Like neurons, however, all oligodendrocyte lineage cells are incredibly sensitive to death ligands, which trigger fully functional apoptotic or necroptotic machinery.

### Extrinsic apoptotic regulation in GBM

Almost all human GBM cell lines express the full extrinsic apoptotic machinery [379, 380]. However, high CASP8 expression has been associated with worse survival given sublethal actions of CASP8 in tumor growth, angiogenesis and cytokine secretion via alterations to NF-κB signaling and nuclear localization, as well as in migration via enhancements to Calpain cysteine protease activities [381], which have key roles in GBM invasion and apoptosis resistance [382–385]. Low levels of cleaved (activated) CASP8 are associated with a more aggressive phenotype and worse overall survival [386]. Reduced expression of CASP8 and FADD are seen in GBM relative to normal brain control, with overexpression reducing proliferation and promoting cell cycle arrest and apoptosis [387].

#### TNFα-TNFR1 signaling

TNF-α acts as a growth factor for GBM cells whereby TNF-α neutralization or gene inhibition substantially reduces U251 GBM proliferation [388]. TNFR1 is highly expressed in GBM relative to low grade gliomas and healthy brain tissue [389] and, along with AKT, represents the major mechanism of NF-κB activation in GBM [390]. Almost half of the commonly used human GBM cell lines are sensitive to TNF-α-induced cell death following cIAP1/2 blockade using SMAC mimetics (SMCs) [133, 141]. cFLIP represents the major mechanism of in vitro resistance in the remaining lines [380]. EGFR inhibition causes significant increases in TNF-α expression, with subsequent signaling through TNFR1 and activation of JNK and ERK resulting in enhanced GBM survival; inhibition of TNF, JNK and/or ERK signaling sensitizes GBM cells to EGFR inhibition both in vitro and in vivo [391, 392]. Notably, combined EGFR and TNF-α inhibition significantly reduces viability of TMZ-resistant (MGMT unmethylated) recurrent human GBM cells, and is as potent as TMZ in MGMT methylated cells [393]. Whether SMC-mediated cIAP1/2 degradation would sensitize to TNF-α induced cell death upon combination with EGFR blockade may be a worthwhile avenue of exploration. Indeed, SMCs significantly enhance colorectal cancer cell death upon concomitant EGFR blockade and TRAIL treatment [394]. Mesenchymal GSCs are enriched for TNF-α receptor superfamily members, as well as NF-κB pathway members; NF-κB controls transcription factors crucial for mesenchymal differentiation. To that end, TNF-α-TNFR1 signaling can induce a proneural-to-mesenchymal phenotype switch of GSCs, with downstream changes in expression of anti-apoptotic genes contributing to resistance to RT. Mesenchymal differentiation and high NF-κB activation are predictive of poor sensitivity to RT in vivo [395]. SMCs are the major chemical agents targeting the extrinsic apoptotic and necroptotic pathways, and their use in preclinical settings for enhancing GBM cell death are summarized in Table 1.

#### FAS-FASL signaling

AKT phosphorylation and consequent inactivation of the FOXO transcription factors result in low FASL expression [100]. Nonetheless, FASL is expressed by the majority of GBM cell lines and primary GBMs, with FASL potentially acting to kill infiltrating lymphocytes [386, 396–398]. Indeed, Ichinose et al. found correlations between areas of high GBM cell and tumor vasculature FASL expression and reduced lymphocyte infiltration [398]. Along these lines, Choi et al. found that FAS signaling increased GBM expression of IL-8, MCP-1 and IL-6, which have roles in T-cell migration and activation [397]. GBM cells express significantly higher levels of FASL, FAS and cleaved CASP8 relative to bulk normal glia [386]. Upregulation of FAS on the 36B10 rat GBM model significantly increases overall survival in vivo, with substantial leukocyte infiltration relative to wildtype 36B10 tumors [399]. Interestingly, high FAS expression has been associated with low overall survival. Disruption of FAS-FASL signaling using the FASL-targeting APG101 fusion protein improved survival rates in phase 2 clinical trials and GBM mouse models, synergizing with RT [400]. Given low FAS expression, GBM cells are resistant to FASL-induced apoptosis [396, 401, 402] and FAS surface expression correlates with FASL cytotoxicity.

Nuclear factor of activated T-cells-1 (NFAT1) is overexpressed in GBM and correlates with FAS and FASL levels. The induction of NFAT1 levels with combined phorbol myristate acetate and ionomycin treatment sensitize U87 and U251 GBM cells to FAS-mediated apoptosis [403]. The FASL decoy receptor DCR3 is expressed on GBM cells, resisting immune-mediated cell death in vivo [404, 405]. Gamma irradiation substantially increases expression of FAS on U87 GBM cells and potently sensitizes to FASL-induced cell death in vitro [257]. Eisele et al. found a trend for higher FAS expression on GSCs relative to more differentiated cells. All GSCs examined expressed CASP8, with little changes in expression relative to differentiated cells. This pattern was also seen in the expression of CASP3, cFLIP, XIAP, BAX, and BCL-2. Interestingly, they found that combination APO010 and TMZ significantly enhanced apoptosis in GSCs moreso than in differentiated cultures [406]. Moderate sensitivity of bulk U87 cells to FAS-induced apoptosis has been found, while GSC-enriched neurosphere cultures exhibited significantly reduced sensitivity, likely a result of observed reductions in functional FAS expression [407]. This suggests GSCs, like their NSC counterparts, are type 2 cells.

#### TRAIL signaling

Most human GBM cell lines are resistant to TRAIL-induced apoptosis despite high expression levels of TRAILR2 (but minimal TRAILR1) expression [379], yet both at substantially higher levels than seen in astrocytes [78, 85]. Expression of TRAILR2 has been found to correlate with survival but not with glioma grade [406]. U87 human GBM cells respond to SMC and TRAIL cotherapy in vivo. This treatment had no effect on normal human astrocytes in vitro and no neurotoxic effects in vivo upon direct injection into brain [138]. Arsenic trioxide increases TRAILR2 expression and sensitizes GBM cells to TRAIL without affecting cFLIP or AKT [338]; other agents acting to similarly increase TRAILR1/R2 also sensitize GBM cells to TRAIL [408].

GSCs are even more resistant to TRAIL-induced apoptosis, with a lack of TRAILR1 and TRAILR2 expression and higher cFLIP levels relative to more differentiated cells. cFLIP is cleaved to its active form in TRAIL resistant GBM cell lines but not in sensitive ones [409]. Interestingly, cisplatin treatment reduces cFLIP and increases TRAILR2 expression, potently sensitizing GSCs to TRAIL and reducing sphere forming capacity in vitro [410]. Reduced NF-κB signaling and consequent cFLIP reductions enhance TRAIL-induced apoptosis [411]. Radiation induces soluble TRAIL expression and secretion by U87 GBM cells, as well as modest increases in TRAILR2 expression [257]. U87 and U251 GBM cells are sensitive to irradiation and/or TRAIL-induced, CASP8-mediated apoptosis. Isolated GSCs from these cell lines are significantly more resistant to either and express significantly lower levels of TRAILR2 and CASP8. cFLIP decreased in sensitive, differentiated cells, but was unchanged in GSCs. NF-κB activation was also significantly higher in GSCs. Exposure to radiation in GSCs also increases expression of TRAILR2. While more differentiated U87 cells show no changes in cFLIP expression following irradiation, GSCs substantially increase cFLIP levels. However, subsequent treatment with TRAIL potently decreased cFLIP in both U87 and U251 GSCs and induced significant CASP8 mediated apoptosis [412]. GBM cell lines that resist induction of cell death by SMC + TRAIL display differences in caspase processing relative to those that respond, with lower pro-CASP8 and BID expression. Non-responders require intrinsic apoptotic cascade stimulation and are sensitized upon BCL-2 blockade, an effect not seen in normal NSCs [413]. Higher levels of MCL-1 were found in TRAIL-resistant GBM cells compared to sensitive. MCL-1 knockdown or inhibition potently sensitized to TRAIL-induced apoptosis, an effect requiring Noxa-mediated disruption of MCL-1:Bim or MCL-1:BAK interactions [414].

#### PEA-15 effects

GBMs show upregulated PEA-15 protein levels relative to normal brain [85]. GBM lines resistant to death receptor stimulation display double phosphorylated PEA-15 capable of inhibiting DISC formation, whereas sensitive lines show only unphosphorylated and single phosphorylated forms [409]. This is consistent with inactivation of PTEN and high activation of AKT observed in GBM cells. Indeed, inhibition of AKT signaling significantly enhances U87, A172 and U251 human GBM cell sensitivity to TRAIL [415]. The role of PEA-15 in GBM may be more pronounced in vivo than in vitro, allowing cells to adapt to more diverse cellular stresses. In vivo, double phosphorylated PEA-15 was found in perinecrotic regions and significantly increased upon exposure to hypoxic and low glucose conditions. Fresh, ex vivo human GBM samples showed significantly higher levels of doubly phosphorylated PEA-15 than common cell lines. Upon glucose withdrawal, PEA-15 phosphorylation is increased in GBM cells, with consequent increases in ERK1/2 phosphorylation and Glucose transporter 3 (GLUT3) expression, each providing further anti-apoptotic effects.

Illustrating a crucial role of PEA-15 in adapting to in vivo microenvironment changes, siRNA knockdown of PEA-15 almost eliminates the ability of U87 GBM cells to form tumors. Interestingly, through modulations of ERK signaling, PEA-15 plays a role in upregulating GLUT3, allowing for enhanced glucose uptake in low glucose environments [416]. Protein levels of PEA-15 have been found to correlate with resistance to TRAIL; resistant cell lines displayed approximately twofold higher levels than sensitive cells. Interestingly, cFLIP levels do not correlate with TRAIL resistance. PEA-15 overexpression in sensitive cells confers resistance. Targeted PEA-15 knockdown using antisense DNA transfection significantly increased the sensitivity of resistant U373 GBM cells to TRAIL alone [379]. Consistent with this, siRNA knockdown of cFLIP sensitizes resistant human U343 GBM cells to SMC and TNF-α or TRAIL treatment [380], suggesting overlapping but slightly different roles in GBM death ligand resistance between PEA-15 and cFLIP, or cell line differences in expression; U343 cells express low PEA-15 and the kinase CAMKII [78]. These strategies are similar to those observed in astrocytes and oligodendrocytes, with more oligodendrocytic GBMs expected to express more cFLIP. Regardless PEA-15 would be able to compensate in vivo to promote survival, suggesting dual targeting of cFLIP and PEA-15 (either directly or via CAMKII or AKT inhibition) may be most effective in maximizing death receptor-based therapies. High TNFR superfamily activity and presence of CASP8 highlights a potential vulnerability to death ligands and the utility of SMCs. The current major roadblock remains a lack of established cFLIP inhibitors. This difficulty stems from similarity of cFLIP DEDs to those in CASP8 and CASP10 [417], although recent work in identifying molecules specific for cFLIP DEDs have shown promising preclinical effects [418]. Dual targeting of cFLIP and PEA-15 (either directly or via CamKII or AKT inhibition) may be most effective in maximizing death receptor-based therapies.

#### Summation

GBMs resemble reactive astrocytes, NSCs and OPCs exposed to inflammatory cues, ‘primed’ for extrinsic apoptotic cell death (astrocytic, OPC-like) but with populations also requiring intrinsic engagement (NSC-like). The heterogeneity in responses seen within a tumor may be explained both by oncogenic changes as well as differences in apoptotic regulation between CNS cell types. GSCs like NSCs, and more differentiated GBM cells resembling reactive astrocytes and primed OPCs coexisting within a single tumor. The use of whole brain lysates as control removes the possibility of examining heterogeneity of apoptotic protein expression between healthy CNS cell types as the differences between NSCs and astrocytes (resistant) and neurons and OPCs/oligodendrocytes (sensitive), especially in CASP8 expression, may be lost. For example, more astrocytic GBMs, if anything like their healthy counterparts, may be highly resistant to death ligands but potentially primed by inflammatory signaling. Alternatively, OPC or neuron-like tumors may be more sensitive, barring inhibitory mutations. GSCs, if anything like NSCs, may also be highly resistant and require therapies that target both pathways. Such considerations may also reveal novel vulnerabilities. Further, a heterogeneity in response to death receptor stimulation is evident, with both type 1 and type 2 responses seen within a single cell line and, likely, a tumor. Type 2 cells with high AKT activation and subsequent high BCL-2 family expression would require significantly stronger apoptotic triggers to effect cell death, or therapies targeting both pathways. Given all this, as well as the incredible plasticity and dedifferentiation capacity of GBM cells, effective treatments will likely require targeting both pathways. Immunotherapies boosting inflammation and ‘priming’ GBM cells for death-receptor mediated death a la reactive astrocytes and oligodendrocytes, combined with IAP-blockade, will likely be most effective when further combined with agents shifting the heavy anti-apoptotic skewing of intrinsic apoptotic members in GBM. Given the toxicity of systemically-delivered TMZ on the immune system [419], this may require precision delivery directly to tumor site should this agent continue to be used. Enhanced AKT signaling contributes to essentially all the noted anti-apoptotic changes.

## Inhibitor of apoptosis family members in the survival of glioblastoma and CNS cells

### NAIP, LIVIN, BRUCE

Neuronal apoptosis inhibitory protein (NAIP, gene symbol BIRC1), LIVIN/melanoma inhibitor of apoptosis protein (ML-IAP; gene symbol BIRC7) and BRUCE/Apollon (gene symbol BIRC6) are IAP family members with minimal research into their function in GBM. We summarize the literature on their involvement in GBM here.

NAIP possesses three BIR domains as in XIAP, cIAP1 and cIAP2 but lacks E3 ubiquitin ligase activity. It shows highest expression in the brain [420, 421] and is a key regulator of CNS cell death via direct CASP3, CASP7 and CASP9 binding [422–424], with reduced levels observed during neurodegenerative conditions [425]. NAIP has been found at decreased levels in GBM relative to normal brain controls [426] and at significantly higher levels than normal astrocytes [427], potentially illustrating GBM intertumoral heterogeneity and/or differences between CNS cell expression levels. High NAIP expression is correlated with poorer prognosis in glioma patients [428]. Increased expression of NAIP is associated with RT resistance of U87 GBM cells [429].

BRUCE is expressed mainly in endocrine tissues, digestive organs and female reproductive tissue, with moderate expression in the brain [420, 421]. BRUCE is an E2 and E3 ubiquitin ligase required for survival during development. BRUCE plays key roles in cell survival via SMAC and CASP9 ubiquitination and inhibition [430, 431] and inhibits autophagy via degradation of microtubule-associated protein 1 A/1B-light chain 3 [432]. It is expressed at higher levels in GSCs than more differentiated SNB19 GBM cells, and its downregulation contributes to enhanced apoptosis [433].

LIVIN/ML-IAP has E3 ubiquitin ligase activity, however its substrates are less well defined relative to XIAP, cIAP1 and cIAP2. It is minimally expressed outside of the placenta, with minimal expression in the adult brain [420, 421]. Two splice variants (LIVINα and LIVINβ) inhibit intrinsic and extrinsic apoptosis by direct CASP3, CASP7 and CASP9 binding [434, 435]. Significant expression is seen in numerous tumor types where high expression of LIVIN is associated with poor prognosis [436, 437], including GBM [438]. HIF-1α (hypoxia inducible factor-1α) directly binds the LIVIN promoter and increases LIVIN levels under hypoxic conditions, promoting resistance to TMZ and RT [439]. U251 GSCs have been found to express significantly higher levels of LIVIN and the LIVINβ splice variant than more differentiated GBM cells, contributing to enhanced GSC resistance to etoposide chemotherapy [440, 441]. LIVIN levels are reduced in U251 GBM cells following TMZ treatment, with the higher levels in GSCs contributing to enhanced resistance. LIVIN levels have also been found to correlate with U251 proliferation rate [442] and multidrug resistance-associated protein levels [443]. Conversely, TJ905 GSCs showed significantly lower LIVIN levels than their differentiated GBM counterparts [444].

### XIAP and Survivin

#### Glioblastoma levels and effects

XIAP (gene symbol BIRC4) and Survivin (gene symbol BIRC5) are expressed at significantly higher levels in GBM than normal astrocytes [338], although XIAP levels are lower when compared to bulk brain tissue [445]. XIAP is the most potent inhibitor of apoptosis of the IAP family while Survivin the weakest as its effects are indirect, notably via stabilization of the anti-apoptotic function of XIAP [446]. Phosphorylation of XIAP by AKT, which is severely overactivated in GBM and other cancers, significantly enhances XIAP protein stability and consequently anti-apoptotic function [446]. Survivin functions primarily in cell division at the cytokinesis stage of daughter chromosome separation and is typically expressed only in T-cells or stem cells [447], with minimal expression in adult brain [420, 421]. The knockdown of XIAP in multiple GBM cell lines sensitizes these cells to resveratrol, vincristine and doxorubicin-induced cell death [448], illustrating a crucial role of XIAP in GBM chemoresistance and that the intrinsic apoptotic cascade is still functional and targetable. Survivin knockdown in GBM cell lines demonstrate increased apoptosis and reduced proliferation characterized by mitotic catastrophe [449]. Survivin also plays roles in GBM resistance to RT [450]. GSCs have high proteasomal activity and expression of Survivin, and consequently are more sensitive to proteasome inhibitors than more differentiated GBM cells and their NSC counterparts. The proteasome inhibitor bortezomib also reduces Survivin mRNA and protein, reducing GSC spheroid growth and stemness, causing cell cycle arrest, and synergizing with TMZ to enhance apoptosis [451].

The small molecule inhibitor of Survivin, YM155, substantially reduces viability of U87 and U251 human GBM cells, impairs homologous recombination, reverses epithelial to mesenchymal transition (and consequently reduces migration), reduces STAT3 activation, and potently sensitizes GBM cells to radiation [452–454]. Consistent with a functional intrinsic cascade, YM155 inhibition of Survivin synergizes with the BH3-only protein mimetic ABT-737 to induce apoptosis through the mitochondrial cascade in numerous human GBM cell lines. As EGFR signaling through PI3K-AKT regulates Survivin expression, U87 cells with upregulated EGFR or bearing the EGFRvIII mutation exhibited enhanced protection against this cotreatment; EGFR inhibition enhanced sensitivity [455]. Recent work has shown that YM155-induced inhibition of Survivin may be secondary to inhibition of other targets, including topoisomerases [456, 457] or crucial components of mitochondrial function [458]. In keeping with high Survivin expression, SurVaxM, an immunogenic modified version of Survivin, has been used to vaccinate against GL261 murine GBM, producing potent CD8^+^ T-cell responses and significantly improved overall mouse survival [459]. This Survivin-targeted vaccine strategy also shows promising clinical results [460].

Among many cancers, including GBM, Survivin is the most highly upregulated IAP relative to adjacent normal tissue [445] likely due to its obligate role in cell division and the high proliferative index of cancer cells versus normal differentiated cells, especially terminally differentiated neurons. In low grade glioma, Survivin has the strongest impact on survival. This disappears in GBM [445]. Conflicting results show GBMs expressing Survivin had significantly shorter overall survival compared with GBMs that showed no expression. Further, high Survivin expression was associated with a more aggressive phenotype [461]. Using the Chinese glioma genome atlas, high Survivin was associated with poor prognosis [462]. Survivin was significantly increased in GBM relative to control brain tissue. microRNA-218, which reduces Survivin mRNA and protein levels, consequently reduces GBM proliferation, survival, invasion and migration [462]. More recent TCGA analyses show no such correlation, however [445, 463, 464]. The prevailing finding appears to be that Survivin increases with glioma grade and is associated with worse survival [406]. Individual study differences may be accounted for by the disease heterogeneity.

#### CNS levels and effects

NPCs have been found to express high levels of cIAP1 and Survivin, with cIAP1 able to suppress CASP3 activation. The knockdown of cIAP1 sensitizes NPCs to TRAIL [465]. Human embryonic NPCs expressing high TRAILR2 and low CASP8 are resistant to TRAIL. Administration of actinomycin D, which uniformly blocks transcription, can sensitize NPCs to TRAIL-induced apoptosis. Subsequent cell death was found to require both CASP8 and CASP9 [465], which suggests that NPCs are type 2 cells. Given the role of XIAP in determining the necessity for BID cleavage, this would then suggest a high XIAP to caspase ratio. Indeed, under homeostatic conditions XIAP is the second highest expressed IAP in NSCs behind Survivin [466]. XIAP plays a role in preventing apoptosis upon exposure to differentiation cues, especially in neurotrophin receptor interacting MAGE (NRAGE) to bone morphogenetic protein receptor 1α (BMPR-1α) signaling, wherein XIAP can bind both NRAGE and BMPR-1α through its RING domain and prevent retinoic acid, NRAGE, and BMP-induced cell death, as well as activate NF-κB via a XIAP-Table 1-TAK1 complex [467–469].

In NPCs isolated from the mouse SVZ, Survivin expression was found to be substantially higher than in total brain control [470], in keeping with a higher mitotic index and more DNA replication. Survivin is critical for normal CNS development; embryos with gene disruptions display an underdeveloped CNS characterized by substantial neuronal apoptosis [471, 472]. The level of Survivin in NPCs decrease with age, consistent with reductions in proliferation, a consequence of reduced astrocyte-initiated WNT signaling [473]. Granulocyte-macrophage colony stimulating factor (GM-CSF) and its receptor are expressed in the CNS, and it is neuroprotective for embryonic mouse NPCs. This action is via both upregulation of BCL-2 and BCL-xL, and (via PI3K-AKT, MAPK-ERK, and NF-κB signaling) cIAP1 and Survivin [474]. The SOX2 transcription factor is widely expressed in NSCs [475] and is required for homeostasis, self-renewal (through EGFR signaling [476]), and survival, and can directly regulate Survivin expression. In fact, among all IAPs, Survivin protects NSCs from SOX2 deficiency-induced apoptosis [466], and both SOX2 and Survivin are substantially reduced during normal aging and neurodegenerative conditions associated with NSC loss [472, 473, 477–479]. Further, zebrafish NPCs are protected during embryogenesis by hypoxia-inducible factor 2α (HIF-2α)-induced Survivin upregulation [480]. Survivin also directly interacts with and stabilizes β-catenin, elucidating a role in maintaining stem cell pluripotency. Survivin increases during NPC proliferation [471, 481] and decreases during the process of neuronal differentiation [482], illustrating a vital role in neurogenesis and CNS injury responses. Thus, the anti-apoptotic effects of Survivin are indirect through stabilizing and enhancing XIAP and cIAP1/2 functions, primarily playing proliferation-enhancing roles.

Neurons display a high XIAP to caspase ratio [483], with support from glial cells increasing neuronal XIAP and BCL-2 [484]. The cumulative effect of intrinsic pathway alterations in neurons means that the ability of XIAP to prevent apoptosis is significantly enhanced. Reduced activation cues due to low APAF-1 impede apoptosome formation, limiting CASP9 activity and, consequently, CASP3 and CASP7 activation. Low levels of said effector caspases further add to the ability of XIAP to shut down cell death. Expression of these caspases can be induced as a result of injury or in the context of neurodegeneration [485]. This extensive XIAP inhibition allows for alternative caspase functions, such as regulating neurite extensions, facilitating neuroplasticity [486, 487] and modulation of axon degeneration [191, 488], cumulatively preventing cell death following axotomy. CASP3 and CASP9 are involved in axon pruning, with XIAP regulation necessary to maintain sublethal activity [192]. XIAP is further involved in neuronal signaling and survival via its ability to prevent excessive calcium accumulation [489]. In sciatic nerve lesion models, the IAPs (especially XIAP and cIAP1/2) were found to be essential for cell survival, even without neurotrophic factor input [490].

NGF significantly upregulates neuronal XIAP, cIAP1/2 and Survivin mRNA expression [189, 190] and reduces active CASP3 levels via lysosomal degradation. NGF inhibits CYCS loss from the mitochondria via PI3K alterations, regulating BH3-only protein expression [189, 190]. NGF withdrawal consequently increases CASP3 activation and reduces XIAP through proteasomal degradation [191]. Among other common neurotrophic factors essential for neuronal survival, GDNF exposure increases XIAP expression. XIAP is responsible for the protective effects of GDNF, and this is reliant on PI3K signaling. While neuronal injury results in reduced expression of XIAP, exposure to GDNF completely prevents this [491]. In rat cerebellar neurons, the IAP levels vary depending on age. From postnatal day 0 through day 25, cIAP2 and Survivin levels decrease while cIAP1 levels remain constant [492]. XIAP levels substantially increase. XIAP is the most highly expressed IAP and plays the most critical role in neuron survival against a variety of insults and injuries [492–509], including oxidative stress via its role in increasing expression of mitochondrial antioxidants [510]. Interestingly, in retinal ganglion neurons, cIAP2, XIAP and Survivin levels remain constant during aging with a general trend towards decreasing expression levels, while cIAP1 levels are significantly reduced, leading to TRAF2 accumulations, impaired NF-κB signaling and increased susceptibility to cell death [511]. Such age-related decreases in IAP levels may set the stage for neurodegenerative disorders or exacerbate the IAP inhibitory effects of certain neurodegenerative pathologies, such as Alzheimer’s disease. Given that neurons do not divide, the primary function of Survivin is presumably stabilization of XIAP and consequently apoptosis resistance, with increased expression during maturation, following injury or exposure to toxic insults, and under hypoxic and ischemic conditions [190, 512–518].

Little information is available on IAP expression in astrocytes outside of comparisons to astrocytomas. Excitotoxicity causes reactive astrocytes to upregulate Survivin and, minimally, cIAP2. Survivin upregulation is implicated in CASP3 inhibition, not proliferation [519], and likely result from increased STAT3 and NF-κB signaling, common responses to excitotoxicity [520, 521]. Survivin is expressed at relatively low to undetectable levels in resting astrocytes [420, 522–524] but increases following: traumatic brain injury, preventing cell death [513, 518, 525]; in reactive astrocytes responding to intracerebral hemorrhage, promoting cell growth and survival [524]; in the context of Theiler’s murine encephalomyelitis infection, wherein it inhibits CASP3 [526]; and in astrocytes infected with JC virus (which causes demyelination within the CNS) [527]. The increased proliferation noted during astrogliosis is consistent with the function of Survivin in cell division. While XIAP was not examined in these studies, the noted increases in Survivin and attenuated apoptotic responses are likely due to cooperation between the two IAPs. Normal human astrocytes express low levels of XIAP [528]. In response to hypoxia and hypothermia, astrocytes significantly upregulate BAX, BCL-xL, FADD and Survivin transcription and protein levels, as well cleaved CASP3 protein [529]. S100B, which acts as a neuroprotective factor at low concentrations released during astrogliosis, acts via increasing MAPK-Erk signaling [530], with consequent downstream BCL-2 and XIAP increases [484]. cIAP1/2 and XIAP inhibition using SMAC mimeticss does not alter astrocyte resistance to TRAIL, even at very high doses [138].

### cIAP1 and cIAP2

#### Glioblastoma levels and effects

In analyzing TCGA data of patient GBM samples, cIAP2 (gene symbol BIRC3) was the only IAP whose differential expression correlated with overall survival. High cIAP2 was associated with apoptosis resistance and significantly poorer prognosis. Further, cIAP2 levels increased dose-dependently following standard treatments (RT and TMZ), and recurrent tumors showed significantly higher levels than primary. Survivin was found to be increased upon recurrence, and XIAP upon RT [463]. Both of these IAPs are able to stabilize cIAP2 directly or indirectly. The cIAP2 responses to treatment and in recurrence were found to be due to differences in PI3K and STAT3 signaling [463]. Other groups have made similar findings using TCGA analysis, confirming a central role of cIAP2 in GBM prognosis and progression [445]. cIAP2 expression is induced when the NF-κB pathways are activated and is key to resistance of gliomas to TNF-α [531]. cIAP2 is also highly expressed by tumor-infiltrating immune cells, which may confound measurements. Nonetheless, following TMZ treatment, cIAP2 was the most highly upregulated IAP at both transcript and protein levels. Co-treatment with the SMC BV6 significantly increased cell death of TMZ-induced senescent GBM cells [122], illustrating roles for cIAP2 in both cell survival and senescence. In mice deficient in XIAP, compensatory increases in cIAP1 and cIAP2 are observed maintaining cell survival [532, 533]. In GBM, XIAP levels have been found to correlate with cIAP2 levels, but not cIAP1. cIAP2 is significantly increased during gliomagenesis. Direct binding of XIAP to cIAP2 through its RING domain is required for XIAP stabilization of cIAP2, and this stabilizing effect may explain the slower kinetics of SMC-induced degradation of cIAP2 relative to cIAP1 [534]. However, the varying affinities for SMCs to the IAPs, and the associated loss of cIAP1 (a degrader of cIAP2 via E3 ubiquitin ligase activity) can lead to the stabilization of cIAP2 [535, 536]. SMC-induced production of TNF-α and/or activation of NF-κB pathways results in transcriptional upregulation of cIAP2 mRNA levels [537–540] countering drug efficacy in degrading cIAP2.

High levels of hypoxia and necrosis are associated with mesenchymal phenotype, aggressive disease, and poor survival. cIAP2 is found at the highest levels in mesenchymal GBMs. NF-κB signaling mediates the mesenchymal transformation as well as cIAP2 expression. Hypoxia also increases cIAP2 expression, with HIF-1α binding at the BIRC3 promoter; cIAP2 inhibition of caspase activation subsequently promotes survival under hypoxic conditions and upon exposure to RT [464]. Other studies have shown hypoxia does not affect IAP expression, with a relatively equal distribution independent of oxygen levels. Interestingly, IAP inhibition using SMCs was enhanced under hypoxic conditions. However, whereas under normoxic conditions IAP inhibition increases GSC astrocytic differentiation, similar treatments in hypoxia did not alter stemness [541]. Increases of cIAP2 levels during hypoxia depend on concurrent increases in XIAP. TNF-α also specifically increases cIAP2 in GBM cells [534]. Between low grade glioma and GBM, only cIAP2 and XIAP showed significant upregulation. In low grade glioma, high cIAP1/2 were associated with poor survival, while only cIAP2 had prognostic value in GBM. cIAP2 was found to promote malignant progression [542]. IAP blockade and subsequent alterations in NF-κB signaling affect GSC differentiation towards a more astrocytic fate, with reduced stem cell markers; no such effect is seen on normal NSCs [543]. cIAP2 has been found to enhance stemness features and maintain stem cell self-renewal of GBM cells in vitro via alterations to BMP4 signaling, increasing neurosphere forming potential and upregulating canonical cancer stem cell markers. In vivo, cIAP2 overexpression accelerates tumor initiation and progression and significantly reduces overall survival time [544].

#### CNS levels and effects

Levels of cIAP1 are significantly higher in the mature CNS than cIAP2 [420]. In NSCs under homeostatic conditions, cIAP2 is expressed at the lowest levels behind Survivin, XIAP and cIAP1 (in this order) [466]. Among 34 examined anti-apoptotic genes following treatment of NPCs with FASL, no changes in expression were noted in Survivin, cIAP1, or XIAP, but cIAP2 levels increased sevenfold at both the mRNA and protein levels [254]. Developmental changes in neuronal IAP levels demonstrate reductions in cIAP2 associated with maturation following the completion of migration and synaptogenesis [492]. Hyaluron (HA), a major component of the brain extracellular matrix/perineuronal net, substantially increases neuronal NF-κB signaling and cIAP2 levels. Consequently, HA can protect neurons in vitro from hydrogen peroxide cytotoxicity. Further, HA induces BDNF and EGF production by astrocytes, acting as added neuroprotective factors 545]. Low cIAP2 in normal brain and in reactive astrocytes [546] suggests a reliance on cIAP1 and little induction following death ligand or inflammatory cytokine exposure. Indeed, in familial amyotrophic lateral sclerosis models, reactive astrocytes significantly increase cIAP1 levels [547]. Astrocytes also rely on PEA-15 for resistance to TNF-α - exposure in the absence of PEA-15 induces substantial apoptosis within 24 h [71]. This is in keeping with the noted ability of TNF-α to induce astrogliosis instead of cell death.

Interestingly, OPCs also express significantly higher cIAP2 than differentiated oligodendrocytes [351], an effect similar to the reduction of cIAP2 levels observed as neurons mature. In the context of spinal cord injury, the experimental cytokine treatment leukemia inhibitory factor (LIF) is protective against IFN-γ and TNF-α, acting on microglia to release IGF-1 [548] which signals for oligodendrocytes to upregulate cIAP-2 via AKT [549]. Following traumatic brain injury, which engages both intrinsic and extrinsic apoptotic pathways, levels of cIAP1/2 in oligodendrocytes increased within 1 h, with cIAP2 elevations sustained for longer than cIAP1, potentially compensating for low XIAP [506]. LIF, which activates PI3K-AKT signaling through the LIF Receptor β, significantly reduces demyelination and oligodendrocyte cell death following spinal cord injury. LIF induces significant increases in cIAP2 two weeks post-injury. This increased cIAP2 was associated with greater cIAP2-CASP3 interactions and reduced levels of cleaved CASP3. Mature oligodendrocyte apoptosis was consequently reduced, explaining the reduced demyelination following LIF treatment [550]. NF-κB signaling and cIAP2 levels are increased following compression spinal cord injuries correlating to degree of compression [545]. In TNFR1-/- and TNFR2-/- mice, cIAP2 is reduced following spinal cord injury because of decreased NF-κB signaling. Interestingly, functional rescue in TNFR2-/- mice was noted, likely due to characteristic delayed cIAP2 increases reducing apoptosis. In WT mice, cIAP2 is increased, representing an anti-apoptotic response to spinal cord injury [551].

#### Summation

Regarding XIAP, GBM tumors most resemble NSCs and reactive astrocytes, although given heterogeneity single cell analyses will likely reveal more oligodendrocytic cells expressing low XIAP. Given the crucial role of XIAP in GBM chemoresistance, it is not surprising that knockdown of XIAP sensitizes GBM to chemotherapy and illustrates the intrinsic apoptotic cascade is still functional and targetable, with XIAP foundational to the heavy anti-apoptotic skewing. This further shows the promise of IAP-targeting SMCs in the treatment of GBM. GBM cells are more like NSCs and reactive astrocytes than oligodendrocytes in their Survivin expression profile. Assuming a heterogeneous Survivin expression profile based on differentiation states within a tumor, it holds that like the normal CNS, GSCs would harbour the highest Survivin levels. Targeting these cells has long been a research goal given their fundamental roles in gliomagenesis, therapy resistance and recurrence. Survivin-targeted therapies may achieve it. Unlike NSCs and astrocytes, GBM cells appear more sensitive to cell death triggered by Survivin inhibition. GBMs resemble NSCs and OPCs exposed to inflammatory cues and responding to injury regarding increased/high cIAP2 responses/levels.

## Conclusion

GBM is characterized by intra- and intertumoral heterogeneity in gene expression profiles, migratory behaviours and responses to treatment. Consistent with this, heterogeneity in cell death responses exist between tumors and between cells within a tumor. Improved sequencing techniques and tailored, personalized medicine approaches are likely to be key aspects of successful treatment for this highly lethal and treatment refractory cancer for which no significant improvements in survival have been achieved in decades.

GBM cells have been found to recapitulate many features of normal CNS development and injury responses. Given this, as well as intensive research focus on enhancing GBM drug delivery through BBB opening, intracerebroventricular drug delivery or surgical bed implantation of chemotherapeutic wafers, understanding the cell death mechanisms used by normal CNS cells is crucial for understanding potential CNS toxicities and personalizing treatments for this highly heterogeneous cancer. As the cell of origin for GBM has not been firmly established, understanding cell death responses by normal CNS cells relative to their transformed counterparts can allow for more informed, personalized treatments tailored to target predominant cell types within a GBM and exploit vulnerabilities resulting from oncogenic transformation while limiting toxicity. To our knowledge this is the first review outlining comparisons between GBM and normal CNS cells in their cell death control mechanisms.

## Data Availability

No datasets were generated or analysed during the current study.
